# Top-down attention and Alzheimer’s pathology affect cortical selectivity during learning, influencing episodic memory in older adults

**DOI:** 10.1126/sciadv.ads4206

**Published:** 2025-06-13

**Authors:** Jintao Sheng, Alexandra N. Trelle, America Romero, Jennifer Park, Tammy T. Tran, Sharon J. Sha, Katrin I. Andreasson, Edward N. Wilson, Elizabeth C. Mormino, Anthony D. Wagner

**Affiliations:** ^1^ Department of Psychology, Stanford University, Stanford, CA, USA.; ^2^ Department of Neurology and Neurological Sciences, Stanford University School of Medicine, Stanford, CA, USA.; ^3^ Wu Tsai Neurosciences Institute, Stanford University, Stanford, CA, USA.

## Abstract

Effective memory formation declines in human aging. Diminished neural selectivity—reduced differential responses to preferred versus nonpreferred stimuli—may contribute to memory decline, but its drivers remain unclear. We investigated the effects of top-down attention and preclinical Alzheimer’s disease (AD) pathology on neural selectivity in 166 cognitively unimpaired older participants using functional magnetic resonance imaging during a word-face/word-place associative memory task. During learning, neural selectivity in place- and, to a lesser extent, face-selective regions was greater for subsequently remembered than forgotten events; positively scaled with variability in dorsal attention network activity, within and across individuals; and negatively related to AD pathology, evidenced by elevated plasma phosphorylated Tau_181_ (pTau_181_). Path analysis revealed that neural selectivity mediated the effects of age, attention, and pTau_181_ on memory. These data reveal multiple pathways that contribute to memory differences among older adults—AD-independent reductions in top-down attention and AD-related pathology alter the precision of cortical representations of events during experience, with consequences for remembering.

## Teaser

Task-evoked dorsal attention network activity and AD pathology influence memory formation in cognitively unimpaired older adults.

## INTRODUCTION

The ability to remember experiences (i.e., episodic memory) is central to effective living, as memories inform understanding of ongoing events, shape thought, and guide decisions and action (*1*). Episodic memory declines later in the lifespan, concurrent with alterations in brain structure and function (*2*, *3*) related to (*4*–*6*) and independent of (*7*, *8*) early Alzheimer’s disease (AD) pathology. A key neural substrate of episodic memory is the representation of event features as they unfold. Notably, some cortical regions preferentially process specific types of information (*9*)—e.g., the fusiform face area (FFA) differentially responds to faces, while the parahippocampal place area (PPA) is differentially tuned to scenes. Neural selectivity refers to the extent to which neurons or cortical regions respond preferentially to specific stimuli or cognitive processes and is thought to yield distinct neural representations of event features that facilitate memory encoding and retrieval. In aging, neural selectivity declines—a phenomenon known as neural dedifferentiation—whereby neural responses become less distinct across different stimulus types (*10*–*17*). While this decline in selectivity is thought to contribute to age-related cognitive decline (*10*, *11*, *15*, *18*, *19*), the mechanisms driving these changes remain unclear. Here, we use functional magnetic resonance imaging (fMRI) along with biomarkers of preclinical AD pathology in a large sample of cognitively unimpaired older adults to identify mechanisms and pathways underlying altered neural selectivity during episodic memory formation in human aging.

In humans and other primates, top-down attention is known to influence neural selectivity during perception (*20*–*23*) and memory encoding (*24*–*26*) and predicts subsequent memory performance (*25*, *27*–*29*). For example, top-down attention reduces the overlap in the neural populations representing attended versus unattended objects, increasing neural selectivity in ventral visual cortex (*20*). Extant data further indicate that top-down attention is diminished in older relative to younger adults (*30*, *31*) and that age-related differences in attention partially account for age-related declines in episodic memory (*32*, *33*). However, across older adults, there is marked variability in attention-dependent performance, including on selective attention and sustained attention tasks (*34*, *35*), and in episodic memory (*6*, *36*). Here, we hypothesize that individual differences in the strength of neural selectivity across cognitively unimpaired older adults may partially reflect differences in top-down attention, with consequences for subsequent memory.

Hallmark pathological features of AD—tau and amyloid-β (Aβ) proteins—begin accumulating decades before the emergence of clinical symptoms of dementia (a stage referred to as preclinical AD) (*37*, *38*). Amyloid deposition is typically widespread throughout many neocortical association areas, including in frontoparietal cortical areas (*39*), whereas abnormal tau accumulation begins in the medial temporal lobe (*40*, *41*), ventrolateral temporal cortex, and retrosplenial/posterior cingulate cortex (*42*–*44*). This initial pattern of tau deposition is observed during the preclinical AD stage and is associated with reductions in memory performance (*41*, *42*, *45*, *46*), raising the possibility that preclinical AD-related memory decline in aging may be partially mediated by changes in neural selectivity. Along these lines, Maass *et al.* (*47*) showed early tau positron emission tomography (PET) burden was negatively associated with neural selectivity in the posterior-medial network (i.e., place-selective regions) in 50 cognitively unimpaired older adults. Here, we leverage biofluid biomarkers of preclinical AD to explore the relations between AD pathology, neural selectivity, and memory in a large sample of cognitively unimpaired older adults.

Moreover, to the extent that there are links between differences in attention and neural selectivity in older adults, an additional open question is whether these links relate to preclinical AD processes. On the one hand, extant data indicate that the locus coeruleus, one of the earliest brain regions affected by tau protein accumulation (*48*, *49*), plays a crucial role in arousal and alerting (*50*) and modulates multiple aspects of attentional function through interactions with the dorsal attention network (DAN), a key network supporting top-down attention (*35*, *51*–*55*). Consequently, top-down attention may be influenced by early AD pathology (*54*, *56*). On the other hand, age-related changes in neural systems of attention may occur through AD-independent processes, including processes that give rise to structural (*57*–*59*) and functional (*60*, *61*) changes to the DAN. Hence, we examine whether early AD pathology affects neural selectivity in cognitively unimpaired older adults through disease-related changes in task-evoked DAN activity, with consequences for later remembering or whether any links between AD pathology, neural selectivity, and memory represent a distinct pathway from that related to attention.

**Fig. 1.**
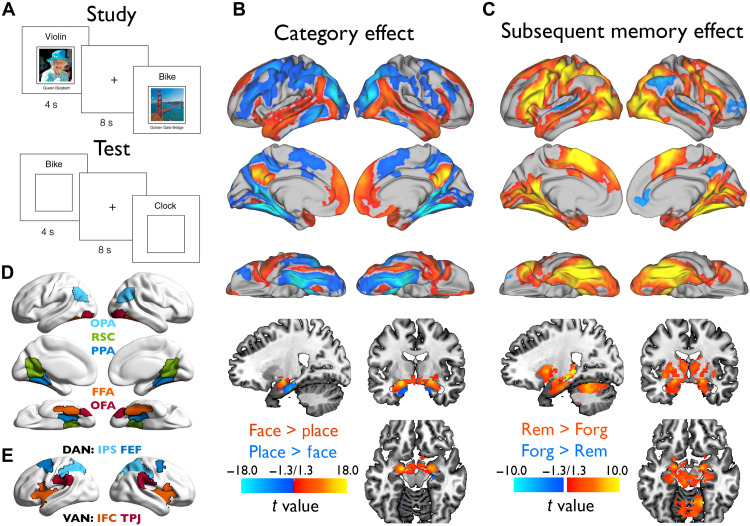
Experimental design, group-level statistical maps, and ROIs. (**A**) Structure of study (encoding) and test (retrieval) trials in the word-picture associative memory task. (**B**) Group-level category effects (face-related versus place-related neural activity) during word-picture study. (**C**) Group-level SMEs depicting differential activation on word-picture encoding trials for which the association was subsequently remembered (Rem) versus subsequently forgotten (Forg) at test. The colored brain areas in (B) and (C) represent significant results after family-wise error (FWE) and threshold-free cluster enhancement (TFCE) whole-brain correction. The correction was applied separately for the left hemisphere, right hemisphere, and subcortical regions, with a threshold of −log₁₀(α/*N*) = −log₁₀(0.05/3) = 1.7782. These significant regions were then mapped onto the *t*-statistical maps. (**D**) Predefined place- and face-selective ROIs. (**E**) Predefined frontoparietal DAN and ventral attention network (VAN) ROIs.

To address these knowledge gaps, we leverage a large sample (*n* = 166) of cognitively unimpaired older adults (60 to 88 years) from the Stanford Aging and Memory Study (SAMS) who performed a word-face and word-place associative memory task (Fig. 1A) concurrent with high-resolution fMRI (*6*, *36*). Here, we focus on category-level neural selectivity during memory formation, defined as (i) univariate differences in blood-oxygen-level-dependent (BOLD) signal between preferred versus nonpreferred stimuli in face- and place-selective regions of interest (ROIs) and by (ii) a trial-level metric of selectivity in these ROIs computed using pattern similarity analysis (PSA). Next, we compute (i) univariate activation differences between subsequently remembered versus forgotten trials [i.e., subsequent memory effects (SMEs)] in the DAN, yielding an indirect memory-relevant neural index of top-down attention (*35*, *53*, *55*, *62*), and (ii) a trial-level measure of task-evoked DAN activity restricted to subsequently remembered trials, which controls for possible attention-independent memory effects on DAN activity. Last, in a subset of participants, we assay early AD pathology using plasma (*n* = 138) and cerebrospinal fluid (CSF) (*n* = 115) phosphorylated Tau_181_ (pTau_181_) and Aβ_42_/Aβ_40_. Analyses examine whether trial-level and participant-level measures of neural selectivity during encoding predict subsequent memory; how neural selectivity varies with age, DAN activation, and AD biomarkers; and, using structural equation modeling (SEM), whether multiple pathways account for memory variability among cognitively unimpaired older adults.

## RESULTS

### Associative memory behavior

Participants underwent fMRI during both associative encoding (data reported here) and associative retrieval (*6*, *36*). During retrieval scans, participants encountered studied and novel words, were instructed to attempt to recall the specific associate for studied words, and responded by indicating whether each word had been paired with a face, place, was old (but could not recall the associate), or was novel (see Materials and Methods). To further assess participants’ ability to recall the specific exemplar paired with each word, they completed a postscan cued recall test measuring successful retrieval of the associated image.

Associative *d*′ (*Z*_associative hit rate_ − *Z*_associative false alarm rate_) assessed in-scanner memory performance and computed overall (i.e., pooled across categories) and separately for face (i.e., word-face associations) and place (i.e., word-place associations) memory, which were highly correlated (fig. S1A). Consistent with our previous findings (*6*, *36*), associative *d*′ declined with age (overall: β = −0.044, *P* < 0.001; face: β = −0.030, *P* = 0.002; place: β = −0.044, *P* < 0.001; fig. S1, B and C). A marginal age × category interaction suggests that age may affect memory for places more than for faces (*F*_1,164_ = 3.477, *P* = 0.064, partial η^2^ = 0.02). Similarly, proportion correct recall of the specific exemplars (*N*_exemplar correct_/*N*_all old_) during the postscan test decreased with age (overall: β = −0.011, *P* < 0.001; face: β = −0.011, *P* < 0.001; place: β = −0.011, *P* < 0.001; fig. S1D) and strongly correlated with in-scanner associative *d*′ (overall: β = 0.170, *P* < 0.001; face: β = 0.159, *P* < 0.001; place: β = 0.206, *P* < 0.001; fig. S1E). In addition, a trial-wise logistic regression analysis revealed that making an in-scan associative hit was a significant predictor of successful postscan specific exemplar recall (overall: β = 1.578, *P* < 0.001; face: β = 1.646, *P* < 0.001; place: β = 1.710, *P* < 0.001; fig. S1F). These findings suggest that associative hits during the scanned memory test likely often included recall of the specific face or place association. Nevertheless, posttest recall performance was quantitatively lower, due in part to a substantially longer retention interval, change in study-test context, and greater retroactive interference. For this reason, our primary analyses define subsequent memory based on in-scan memory performance, rather than posttest outcomes.

### Category and SMEs

Contrasting word-face versus word-place encoding trials revealed well-established neural category effects (Fig. 1B and table S1). Specifically, activation was higher on face trials in bilateral occipital face area (OFA), FFA, precuneus, superior temporal sulcus, anterior temporal lobe, medial prefrontal cortex, and amygdala, among other regions, whereas activation was higher on place trials in bilateral occipital place area (OPA), PPA, retrosplenial cortex (RSC), dorsolateral prefrontal cortex (dlPFC), and anterior hippocampus, among other regions.

To identify associative encoding effects, we conducted a subsequent memory analysis (*63*, *64*) that contrasted remembered trials, consisting of word-picture trials that were later remembered (i.e., subsequent associative hits), with forgotten trials, consisting of all other study trials (i.e., subsequent associative misses/incorrect category response, subsequent item hits/“old” response, and subsequent item misses/“new” response). Activation was higher during subsequently remembered than forgotten trials in bilateral ventral visual cortex, frontoparietal components of the DAN, ventral attention network (VAN), and default mode network, and hippocampus, among other regions (Fig. 1C and table S2). Greater activation on subsequently forgotten versus remembered trials was observed in right angular gyrus, precuneus, anterior cingulate cortex/medial prefrontal cortex, dlPFC/frontopolar cortex, and bilateral superior temporal gyrus.

### Subsequently Remembered trials show greater neural selectivity at encoding

**Fig. 2.**
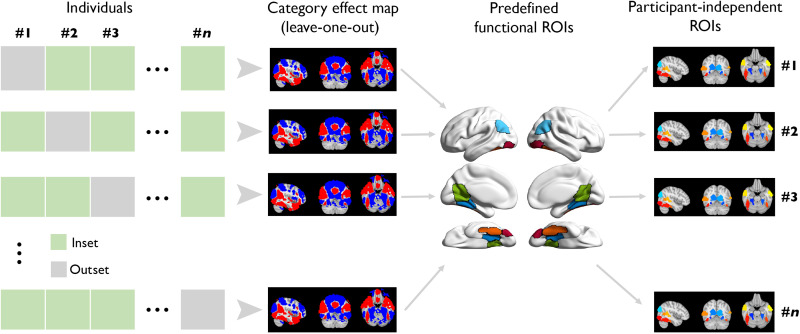
Leave-one-participant-out ROI delineation. For a given participant, we first performed a group-level analysis without the participant to obtain a whole-brain category effect in standard MNI space. A significant category effect map was extracted after controlling for FWE with *P* < 0.05 using TFCE. Next, the resulting whole-brain map was intersected with the predefined functional ROIs, thus yielding study-specific and participant-independent ROIs. Last, we transformed the study-specific ROIs to the given participant’s brain in native space.

For ROI-based analyses, we generated five predefined ROIs known to demonstrate face-selective (FFA and OFA) or place-selective activation (RSC, PPA, and OPA) (Fig. 1D) and using a leave-one-out procedure to ensure statistical independence (Fig. 2 and see Materials and Methods). We then extracted the mean univariate effect (i.e., mean *t* value) in these ROIs when contrasting preferred versus nonpreferred categories (i.e., neural selectivity) as a function of subsequent memory.

Neural selectivity in place-selective (PPA, OPA, and RSC) and face-selective (FFA and OFA) ROIs was greater than zero (*t* = 8.04 to 27.94, *P*_Holm_ < 0.001; fig. S2A), confirming each exhibits greater activity during encoding of words paired with images of the ROI’s preferred category. Moreover, neural selectivity at encoding was higher on subsequently remembered than forgotten trials in both place- and face-selective regions (*t* = 6.62 to 11.02, *P*_Holm_ < 0.001; fig. S2A). When subsequently remembered trials were further categorized into exemplar-specific hits and category-only hits based on the postscan cued recall test (see Supplementary Methods for details), neural selectivity during exemplar-specific hits was significantly higher than during category-only hit trials (*F*_1,940.29_ = 29.288, *P* < 0.001, partial η^2^ = 0.03), with no significant interaction observed across ROIs (accuracy × ROI: *F*_4,940.29_ = 1.285, *P* = 0.274, partial η^2^ = 0.005) (fig. S2B). For the same reasons we described above, the primary measure of neural selectivity for subsequently remembered and forgotten trials was defined on the basis of in-scanner memory performance. Notably, as neural selectivity was strongly correlated across corresponding category-selective ROIs (fig. S2C), we used mean selectivity across the three place-selective ROIs and across the two face-selective ROIs in all subsequent analyses.

### Age-related decreases in neural selectivity

**Fig. 3.**
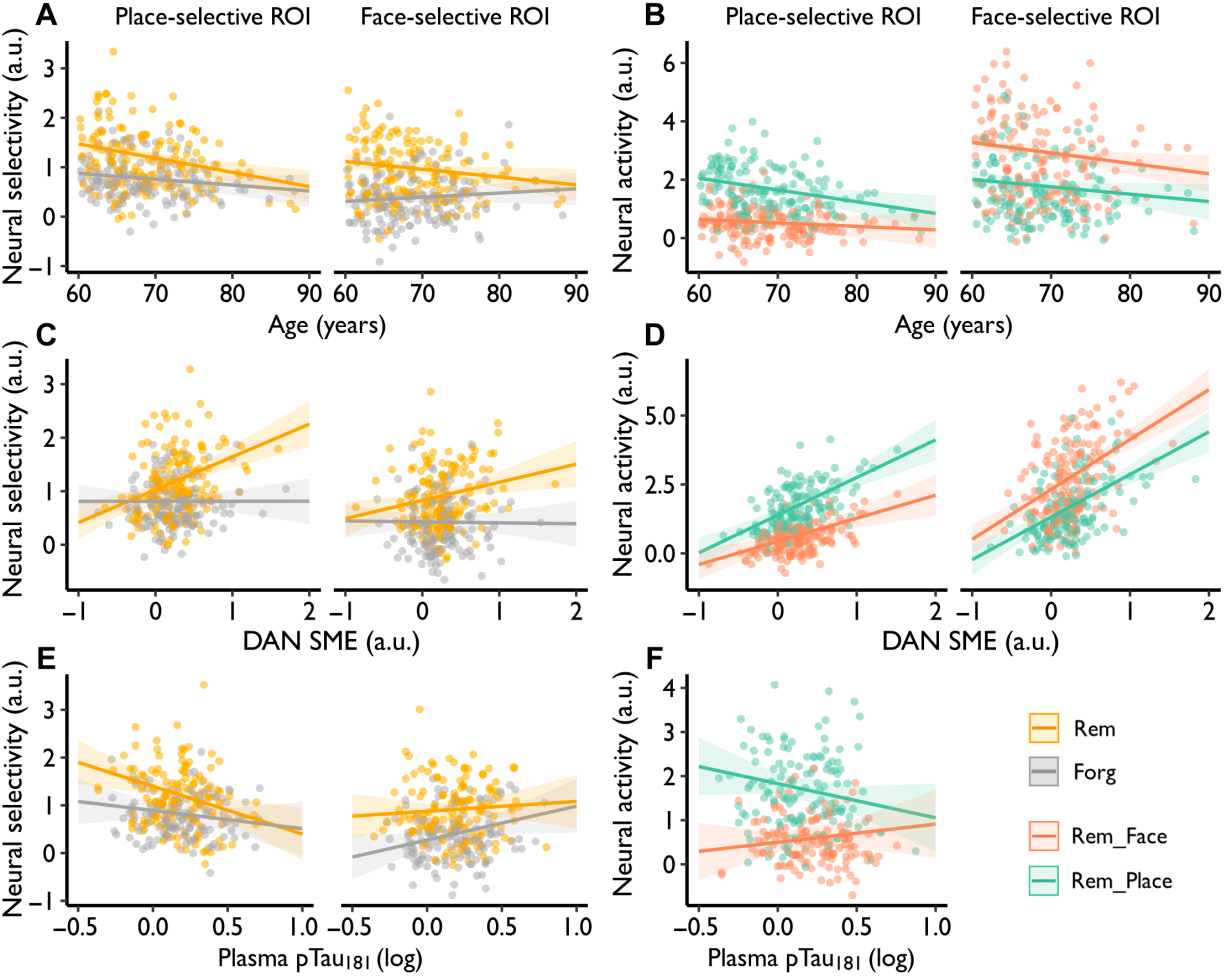
Neural selectivity relates to age, top-down attention, and early AD pathology. (**A**) The age-related decrease in neural selectivity at encoding is greater on subsequently remembered versus forgotten trials. a.u., arbitrary units. (**B**) Neural activity decreased more with age for preferred category than nonpreferred category trials in (left) place-selective but not (right) face-selective regions (analyses restricted to subsequently remembered trials). Sex and years of education were included as nuisance variables. (**C**) Neural selectivity on remembered trials was associated with the SME in frontoparietal nodes of the DAN. (**D**) DAN SME was tightly related to face- and place-related activity on subsequently remembered trials in (left) place-selective and (right) face-selective regions. (**E**) Neural selectivity on remembered trials in (left) place-selective but not (right) face-selective regions significantly declined with plasma pTau_181_. (**F**) Plasma pTau_181_ negatively related to preferred neural activity (i.e., place-related activity, cool color) in place-selective regions. Age, sex, and years of education were included as nuisance variables. Rem_Face and Rem_Place, face-related and place-related neural activity on subsequently remembered trials.

A linear mixed-effect model (LMM) (Fig. 3A and table S3), with age, memory (remembered versus forgotten), and region (face-selective versus place-selective ROI) predictors, revealed that neural selectivity declined with age (*F*_1,152_ = 7.355, *P* = 0.007, partial η^2^ = 0.05) was greater on subsequently remembered trials (*F*_1,456_ = 14.760, *P* < 0.001, partial η^2^ = 0.03) and was greater in place-selective regions (*F*_1,456_ = 8.588, *P* = 0.004, partial η^2^ = 0.02). Moreover, age × region (*F*_1,456_ = 6.756, *P* = 0.010, partial η^2^ = 0.01) and age × memory (*F*_1,456_ = 10.250, *P* = 0.001, partial η^2^ = 0.02) interactions revealed that age-related decreases in neural selectivity were greater in place-selective regions (β = −0.020, *P*_Holm_ < 0.001) compared to face-selective regions (β = −0.004, *P*_Holm_ = 0.508) and on subsequently remembered trials (β = −0.022, *P*_Holm_ < 0.001) compared to forgotten trials (β = −0.002, *P*_Holm_ = 0.758). Last, age-related declines in neural selectivity as a function of memory were similar across place-and face-selective ROIs (age × memory × region: *P* = 0.57, partial η^2^ = 0.0007). Collectively, these results demonstrate that neural selectivity is negatively associated with age, particularly for subsequently remembered trials and place-selective regions.

To determine whether age-related reductions in selectivity reflect attenuation (decreased activity for preferred stimuli) and/or broadening (increased activity for nonpreferred stimuli) (*65*), we examined preferred- and nonpreferred-category activity in face- and place-selective ROIs, restricting analyses to remembered trials (note, selectivity did not change on forgotten trials). An age × category (face versus place activity) × region (face-selective versus place-selective ROI) interaction (*F*_1,456_ = 4.838, *P* = 0.028, partial η^2^ = 0.01; table S4) indicated that the pattern of age-related change in selectivity differed across face- and place-selective ROIs. Follow-up analyses revealed an age × category interaction in place-selective regions (*P* = 0.023), indicating that the age-related decline in neural selectivity was driven by decreased place/preferred-category activity (β = −0.040, *P*_Holm_ = 0.013), without a corresponding increase in face/nonpreferred-category activity (β = −0.012, *P*_Holm_ = 0.381) (Fig. 3B, left). In face-selective regions, the interaction was not significant (*P* = 0.405), face/preferred-category activity decreased with age (β = −0.036, *P*_Holm_ = 0.028), and a qualitatively similar pattern was seen for place/nonpreferred-category activity (β = −0.025, *P*_Holm_ = 0.128) (Fig. 3B, right). Hence, age-related declines in neural selectivity reflect attenuation rather than broadening, with differential reductions in preferred versus nonpreferred activity being more evident in place-selective regions (potentially because face-selective regions show above-baseline activity for both categories).

### Neural selectivity varies with top-down attention network activation

Attenuation of neural selectivity with age and its link to subsequent memory may reflect reduced top-down attentional modulation of category-selectivity activity. To explore this possibility, we extracted the mean univariate effect (i.e., mean *t* value) when contrasting subsequently remembered and forgotten trials (i.e., SME) in frontoparietal regions of the DAN (Fig. 1E and see Materials and Methods), which serves as an indirect neural index of memory-relevant differences in top-down attention (*35*, *53*, *55*, *62*). We observed a significant SME in DAN (*t*_155_ = 9.43, *P* < 0.001) that did not significantly vary with age (β = −0.006, *P* = 0.176; fig. S3A).

An LMM (Fig. 3C and table S5), with top-down attention (DAN SME), memory (remembered versus forgotten), and region (face-selective versus place-selective ROI) as predictors of neural selectivity, revealed an effect of top-down attention (*F*_1,151_ = 9.632, *P* = 0.002, partial η^2^ = 0.06) and a top-down attention × memory (*F*_1,462_ = 18.920, *P* < 0.001, partial η^2^ = 0.04) interaction. These results reflect that neural selectivity increased with greater memory-related top-down attention (DAN SME), and this association was evident for later remembered trials (β = 0.476, *P*_Holm_ < 0.001) but not forgotten trials (β = −0.008, *P*_Holm_ = 0.929). Effects of memory-related top-down attention were similar in place- and face-selective regions (top-down attention × memory × region: *F*_1,462_ = 1.318, *P* = 0.252, partial η^2^ = 0.003; top-down attention × region: *F*_1,462_ = 1.732, *P* = 0.189, partial η^2^ = 0.004). Notably, when age was included in the model, we found that DAN SME (*P* = 0.002) and age (*P* = 0.016) explained unique variance in neural selectivity (table S5). Parallel analysis with frontoparietal regions of the VAN (Fig. 1E), which is linked to bottom-up attention (*53*, *62*), revealed that these effects were specific to DAN-mediated top-down attention rather than reflecting a more general impact of attentional processes on neural selectivity (fig. S3B and Supplementary Results). Thus, during encoding trials that were later remembered, neural selectivity in content-sensitive cortex scaled with memory-related frontoparietal activity in DAN.

To determine whether the association between DAN activity and neural selectivity reflects modulation of activity for the preferred and/or nonpreferred stimulus category, we decomposed selectivity on remembered trials into face and place activity. An LMM revealed a top-down attention (DAN SME) × category (face versus place activity) × region (face-selective versus place-selective regions) interaction (*F*_1,462_ = 6.618, *P* = 0.010, partial η^2^ = 0.01; table S6). Follow-up analyses revealed a DAN SME × category interaction in place-selective regions (*P* = 0.016) but not face-selective regions (*P* = 0.224), which reflects a stronger association between DAN SME and neural activity for place/preferred (β = 1.365, *P*_Holm_ < 0.001) than face/nonpreferred trials (β = 0.838, *P*_Holm_ < 0.001) in place-selective regions, but similar associations for face/preferred (β = 1.811, *P*_Holm_ < 0.001) and place/nonpreferred trials (β = 1.546, *P*_Holm_ < 0.001) in face-selective regions (Fig. 3D). These findings suggest that the enhancement of neural selectivity in place-selective regions by the DAN SME occurs because it increases neural activity for the preferred category more than for the nonpreferred category.

As the DAN SME may reflect differences in top-down attention and attention-independent differences related to memory, we conducted a complementary trial-level analysis that examined the link between activation in the DAN and neural selectivity. Trial-level neural selectivity was defined as the difference between a trial’s pattern similarity to other within-category patterns and its similarity to across-category patterns, computed using multivariate PSA (see Materials and Methods). To control for memory effects, we included only associative hit trials in these trial-level analyses. Using this trial-wise neural selectivity measure, we first observed that neural selectivity for trials from preferred categories was significantly higher than that for trials from nonpreferred categories in both face- and place-selective ROIs (fig. S3C). Moreover, an LMM showed a significant DAN activity × region × category interaction (*F*_1,20936_ = 505.66, *P* < 0.001, partial η^2^ = 0.02). Specifically, trial-by-trial fluctuations in DAN activity were positively related to trial-wise neural selectivity in place-selective regions for place trials (preferred: β = 0.012, *P*_Holm_ < 0.001) but negatively associated with that for face trials (nonpreferred: β = −0.013, *P*_Holm_ < 0.001) (fig. S3D, left). By contrast, in face-selective regions, trial-by-trial fluctuations in DAN activity showed a trend-level positive relationship with trial-wise neural similarity for face trials (β = 0.0008, *P*_Holm_ = 0.073) but negatively associated with that for place trials (β = −0.004, *P*_Holm_ < 0.001) (fig. S3D, right). These outcomes suggest that the above reported relationships between DAN SME and neural selectivity reflect, at least in part, the effects of top-down attention on neural selectivity.

To further investigate the opposing effects observed for preferred and nonpreferred categories in place-selective regions, we separately analyzed the relationship between trial-level DAN fluctuations and within- and across-category similarities. These analyses revealed that for the preferred category (places), higher DAN activity was associated with increased within-category similarity (β = 0.005, *P*_Holm_ < 0.001) and decreased across-category similarity (β = −0.008, *P*_Holm_ < 0.001), whereas the opposite pattern was observed for the nonpreferred category (faces) (within-category: β = −0.006, *P*_Holm_ < 0.001; across-category: β = 0.008, *P*_Holm_ < 0.001) (fig. S3E). Hence, enhanced goal-directed attention may facilitate the integration of place representations within place-selective regions, while simultaneously promoting their differentiation from face representations. Collectively, these outcomes indicate that on subsequently remembered trials, top-down attention increased sensitivity to both preferred and nonpreferred stimuli, affecting neural selectivity particularly in place-selective regions by differentially enhancing responses to preferred stimuli.

### Neural selectivity declines with early AD pathology

Early AD biomarkers of Aβ_42_/Aβ_40_ and pTau_181_, measured in blood plasma (*n* = 138) and CSF (*n* = 115), varied with age (see Supplementary Results). Controlling for age, sex, and education, an LMM predicting neural selectivity (table S7) revealed a plasma pTau_181_ × region (face-selective versus place-selective ROI) interaction (*F*_1,405_ = 14.797, *P* < 0.001, partial η^2^ = 0.04), with higher plasma pTau_181_ related to decreased neural selectivity in place-selective regions (β = −0.690, *P* = 0.011) but not in face-selective regions (β = 0.458, *P* = 0.090) (Fig. 3E). While the plasma pTau_181_ × memory (remembered versus forgotten) interaction in place-selective regions was not significant (*F*_1,405_ = 2.164, *P* = 0.142, partial η^2^ = 0.009), neural selectivity on remembered trials declined with pTau_181_ (remembered: β = −1.0, *P* = 0.004; forgotten: β = −0.379, *P* = 0.268). A similar pattern of decreased neural selectivity in place-selective regions on remembered trials was found for plasma Aβ_42_/Aβ_40_ (fig. S4 and table S7). When decomposing neural selectivity in place-selective regions into activation on place and face trials, an LMM (table S8) revealed a plasma pTau_181_ × category (face versus place activity) interaction (*F*_1,135_ = 10.392, *P* = 0.002, partial η^2^ = 0.07), with activation to place/preferred trials qualitatively declining (β = −0.775, *P* = 0.102) but activation to face/nonpreferred trials qualitatively increasing (β = 0.412, *P* = 0.384) (Fig. 3F). Thus, while selectivity in place-selective regions declined with plasma AD-related proteins, our findings are ambiguous with respect to whether this selectivity change with early AD pathology reflects attenuation and/or broadening. Last, in contrast to neural selectivity, the DAN SME did not vary with plasma (pTau_181_: β = −0.025, *P* = 0.918; Aβ_42_/Aβ_40_: β = 2.608, *P* = 0.229) nor CSF biomarkers (pTau_181_: β = 0.052, *P* = 0.778; Aβ_42_/Aβ_40_: β = −0.786, *P* = 0.577), suggesting that memory-relevant variability in top-down attention is independent of early AD pathology in cognitively unimpaired older adults.

Corresponding analyses of neural selectivity’s relationship with CSF pTau_181_ and Aβ_42_/Aβ_40_ (table S9) revealed similar trends as those observed with plasma (fig. S5), but the effects did not reach significance, perhaps due to the smaller sample size. Consistent with this possibility, restricting analyses to the participants with both plasma and CSF assays revealed qualitatively similar patterns to those reported above (compare Fig. 3E to fig. S6; note the plasma and CSF assays we moderately correlated, Aβ_42_/Aβ_40_: *R* = 0.51, *n* = 103; pTau_181_: *R* = 0.37, *n* = 104; fig. S7).

### Top-down attention and AD biomarkers mediate age-related reductions in neural selectivity

**Fig. 4.**
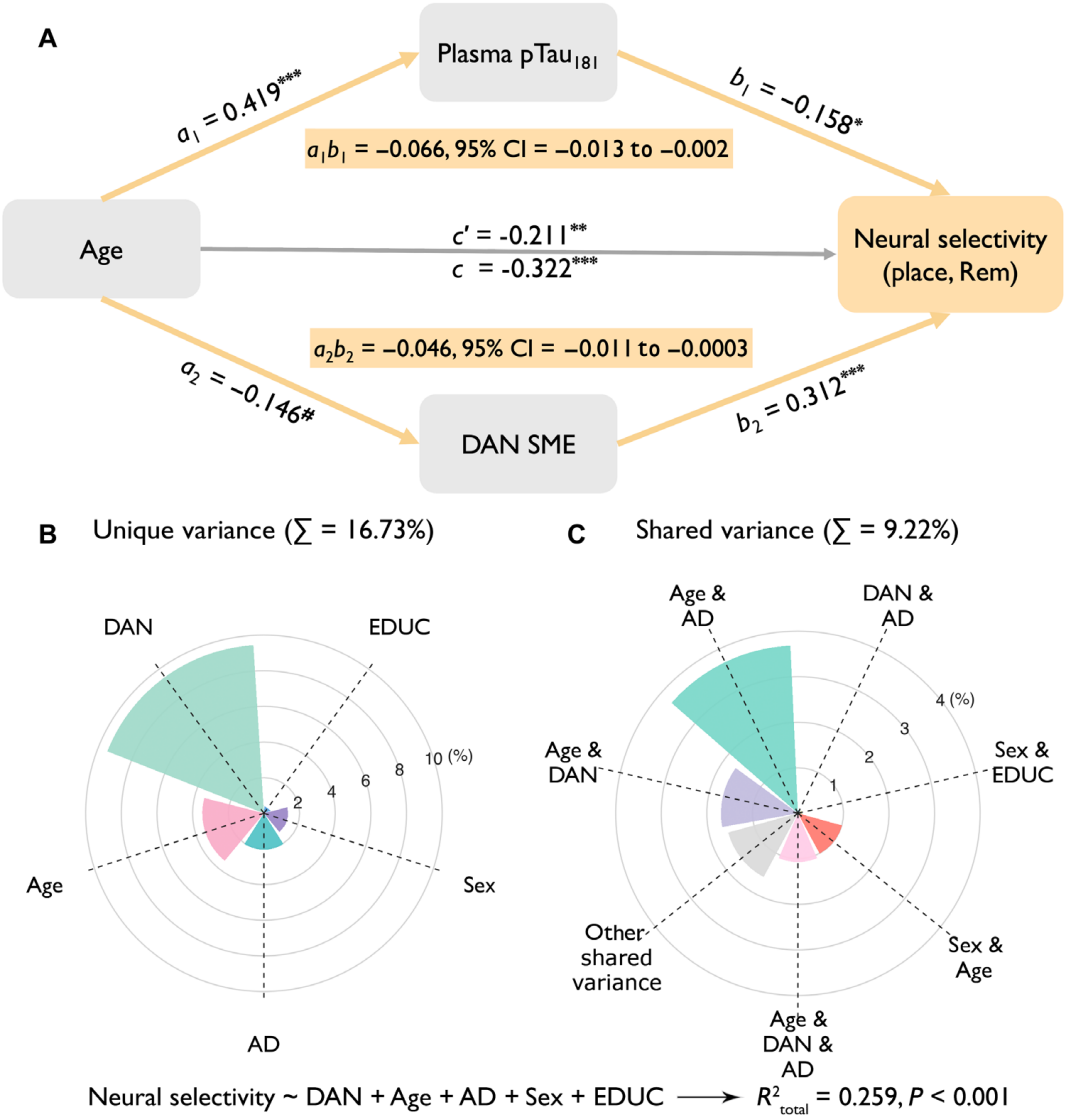
Predictors on neural selectivity. (**A**) Plasma pTau_181_ and DAN SME partially mediated the negative relationship between age and neural selectivity on remembered trials in place-selective regions. Sex and years of education were included as nuisance variables. ^#^*P* = 0.05, *P < 0.05, **P < 0.01, and ***P < 0.001. *c*′ = direct effect; *c* = total effect = *a*_1_*b*_1_ *+ a*_2_*b*_2_ *+ c*′. (**B**) Unique and (**C**) shared explained variances of predictors of neural selectivity. DAN, DAN SME (i.e., SME in DAN); AD, plasma pTau_181_; EDUC, years of education.

Given observed associations between neural selectivity and age, plasma pTau_181_, and DAN activity, we conducted a mediation analysis to determine whether plasma pTau_181_ and DAN SME mediated the effect of age on neural selectivity in place-selective cortex on remembered trials, with sex and years of education as confounding variables. Results revealed that both plasma pTau_181_ and DAN SME partially mediated the negative relationship between age and neural selectivity [indirect1-pTau_181_: *a*_1_*b*_1_ = −0.007, β_standardized_ = −0.066, 95% confidence interval (CI) = −0.013 to −0.002; indirect2-DAN-SME: *a*_2_*b*_2_ = −0.005, β_standardized_ = −0.046, 95% CI = −0.011 to −0.0003; direct effect: *c*′ = −0.022, β_standardized_ = −0.211, *P* = 0.002] (Fig. 4A). Moreover, comparison of the effect sizes in the two indirect pathways revealed that they did not differ from each other (Δβ_indirect1-indirect2_ = −0.002, 95% CI = −0.010 to 0.006), suggesting that early AD pathology and top-down attention have comparable, independent effects on age-related declines in neural selectivity.

To further summarize the unique and shared contributions of age, DAN SME, and plasma pTau_181_ to neural selectivity, we combined these factors in a multiple linear regression model predicting selectivity in place-selective cortex on remembered trials, with sex and education included as covariates. This model explained 25.9% total variance in selectivity (*P* < 0.001; table S10), with DAN SME explaining the most unique variance (*R*^2^_DAN_ = *R*^2^_total_ − *R*^2^_1a_ = 9.4%), followed by age (*R*^2^_Age_ = *R*^2^_total_ − *R*^2^_1b_ = 3.4%), and plasma pTau_181_ (*R*^2^_AD_ = *R*^2^_total_ − *R*^2^_1c_ = 2%) (Fig. 4B). Further, age and plasma pTau_181_ explained shared variance in neural selectivity (*R*^2^_Age&AD_ = *R*^2^_total_ − *R*^2^_2a_ − *R*^2^_Age_ − *R*^2^_AD_ = 3.7%), as did age and DAN SME (*R*^2^_Age&DAN_ = *R*^2^_total_ − *R*^2^_2b_ − *R*^2^_Age_ − *R*^2^_DAN_ = 1.7%) (Fig. 4C). Consistent with their independence, DAN SME and plasma pTau_181_ shared little variance (*R*^2^_DAN&AD_ = *R*^2^_total_ − *R*^2^_2c_ − *R*^2^_DAN_ − *R*^2^_AD_ = 0.1%; Fig. 4C).

### Neural selectivity predicts differences in mnemonic function

**Fig. 5.**
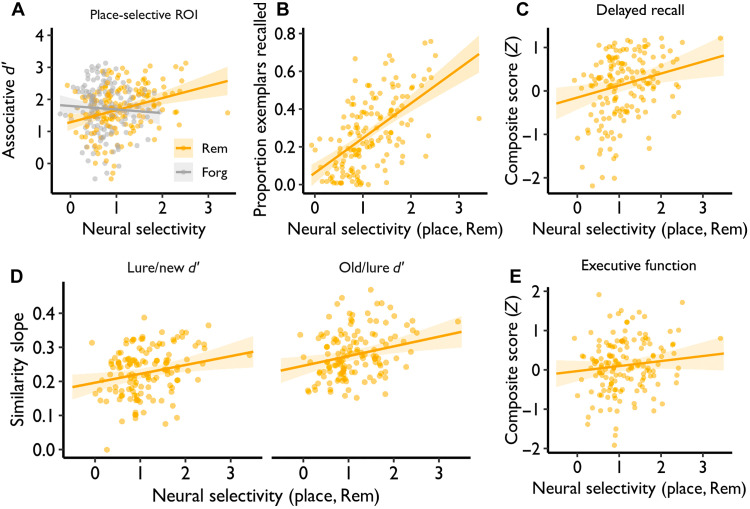
Greater neural selectivity is related to better memory performance. (**A**) Neural selectivity in place-selective regions predicted in-scanner associative memory (overall associative *d*′) on remembered trials. (**B**) Neural selectivity in place-selective regions on remembered trials predicts proportion of exemplar-specific recall on the postscan test. (**C** and **D**) Neural selectivity in place-selective regions on remembered trials predicts out-of-task performance on (C) the delayed recall score during neuropsychological testing and (D) mnemonic similarity task (MST), where the similarity slope on the *y* axis reflects the magnitude of the increase in performance as target-lure similarity moved from high to low. (**E**) Neural selectivity in place-selective regions on subsequently remembered trials was not significantly associated with executive function.

We next asked whether neural selectivity at encoding explains individual differences in within-task and out-of-task cognitive performance, focusing first on selectivity in place-selective cortex on remembered trials and controlling for age, sex, and education. Greater neural selectivity was associated with higher overall associative *d*′ (β = 0.259, *P*_Holm_ = 0.028; Fig. 5A), as well as place (β = 0.366, *P*_Holm_ < 0.001) and face (β = 0.233, *P*_Holm_ = 0.023) associative *d*′ (see Supplementary Results). Moreover, direct comparison revealed a significantly stronger relationship between associative *d*′ and selectivity on remembered than on forgotten trials (Δ*r*_remembered-forgotten_ = 0.337, 95% CI = 0.165 to 0.50; Fig. 5A). Neural selectivity in place-selective regions during subsequently remembered trials also predicted specific-exemplar recall rate on the postscan cued recall test (overall: β = 0.159, *P* < 0.001; face: β = 0.120, *P* < 0.001; place: β = 0.198, *P* < 0.001; Fig. 5B).

We observed similar effects when using a mnemonic similarity task (MST; see Materials and Methods) collected during a different session separated on average by ~4.8 weeks from the fMRI session (Fig. 5D and Supplementary Results), suggesting that the relationship between neural selectivity and memory generalized beyond fMRI task-related and postscan memory performance. Similarly, greater neural selectivity was associated with a higher composite delayed recall score collected, on average, ~4.9 weeks from the fMRI session (β = 0.276, *P* = 0.006; Fig. 5C), whereas selectivity was not significantly associated with a composite executive function score (β = 0.128, *P* = 0.167; Fig. 5E). There was trend-level evidence that neural selectivity differentially predicted individual differences in memory relative to executive function (Δ_delayed recall–executive function_ = 0.196, *P* = 0.062, *n* = 155). Neural selectivity in face-selective regions on remembered trials did not exhibit significant associations with memory performance (see Supplementary Results). Collectively, these data indicate that neural selectivity in place-selective cortex partially explains individual differences in both within- and out-of-task memory behavior.

### Multiple pathways account for memory variability in older adults

**Fig. 6.**
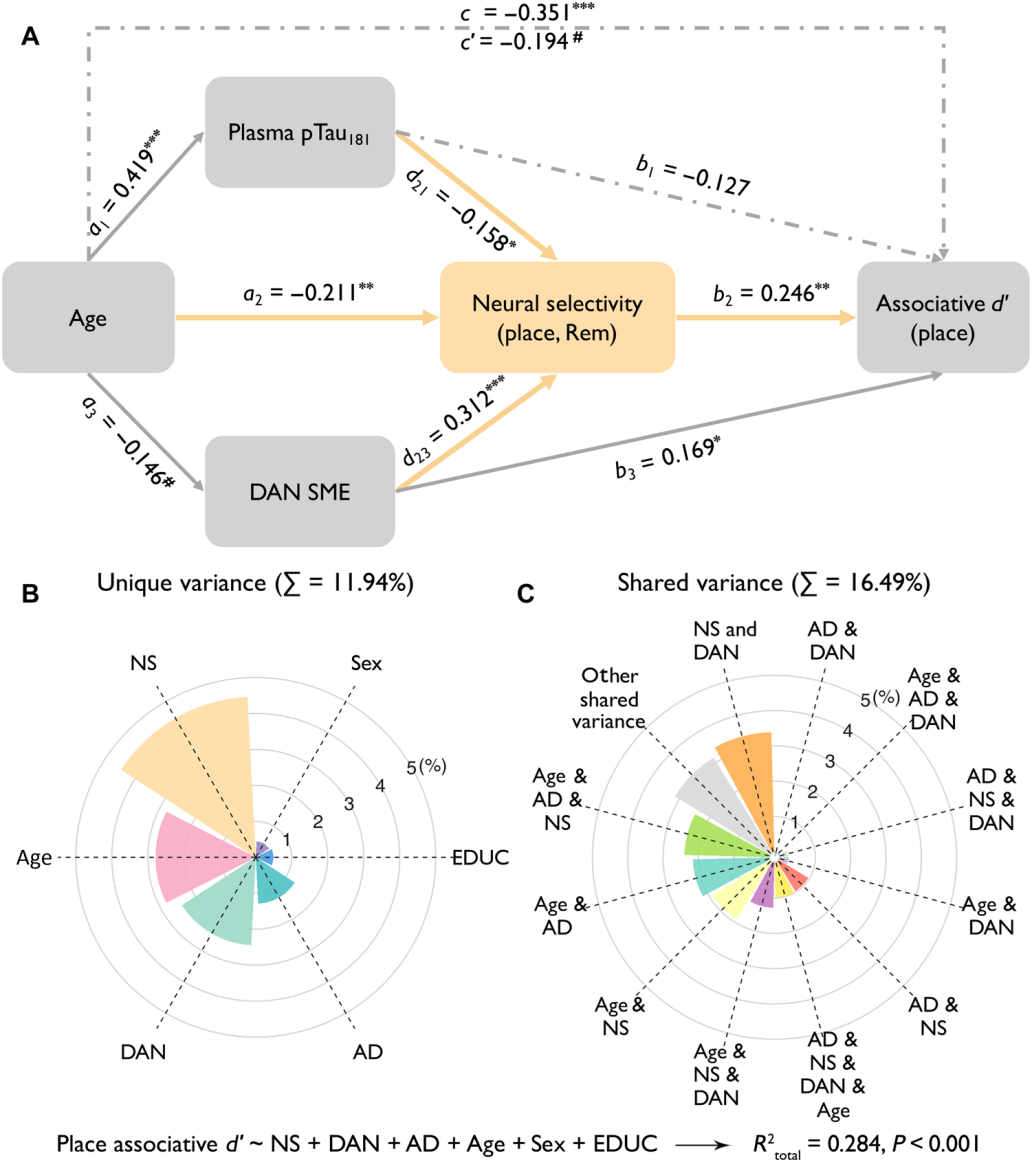
Pathways and predictors on memory performance (i.e., place associative *d*′). (**A**) Structural equation modeling. Solid lines represent significant paths; dash-dotted lines represent nonsignificant paths. The figure shows standardized betas. ^#^*P* = 0.05, *P < 0.05, **P < 0.01, and ***P < 0.001. *c*′ = direct effect; *c* = total effect = *a*_1_*b*_1_ *+ a*_2_*b*_2_ *+ a*_3_*b_3_ + a*_1_*d*_21_*b*_2_ *+ a*_3_*d*_23_*b*_2_ *+ c*′. (**B**) Unique and (**C**) shared explained variances of predictors of place associative *d*′. NS, neural selectivity.

Considering the significant link between neural selectivity at encoding and individual differences in memory, along with the three factors—age, AD biomarkers, and top-down attention—that explain variability in neural selectivity, we developed a SEM to understand pathways from age to neural selectivity to memory. In addition to a pathway from age to neural selectivity to memory, the SEM included two additional pathways through neural selectivity: (i) age ➔ plasma pTau_181_ ➔ neural selectivity ➔ memory performance (AD-related pathway), which tested whether age-related memory decline is explained, in part, by reduced neural selectivity, which, in turn, is explained, in part, by elevated plasma pTau_181_; and (ii) age ➔ DAN SME ➔ neural selectivity ➔ memory performance (attention-related pathway), which tested whether age-related decline in top-down attention (DAN SME) explains, in part, reduced neural selectivity, which, in turn, explains, in part, variance in memory (Fig. 6A and see Materials and Methods). The model included data from 137 participants who had available data for all five factors. Paths not including AD biomarkers were additionally examined in the full sample and replicated the findings in this subsample with all five factors (see Supplementary Results). To simplify the results, we report paths to place associative *d*′ below, which is most related to neural selectivity in place-selective regions on remembered trials (see Supplementary Results for analyses of paths to overall associative *d*′ and face associative *d*′; table S12).

First, the SEM revealed that neural selectivity mediated the relationships between age (*a*_2_*b*_2_ = −0.006, β_standardized_ = −0.052, 95% CI = −0.014 to −0.002), plasma pTau_181_ (*d*_21_*b*_2_ = −0.210, β_standardized_ = −0.039, 95% CI = −0.506 to −0.049), and top-down attention (*d*_23_*b*_2_ = 0.161, β_standardized_ = 0.077, 95% CI = 0.065 to 0.326) with place associative *d*′. These mediation effects were replicated in separate mediation analyses with our full sample for age (table S13) and top-down attention (table S14). These findings demonstrate that, in cognitively unimpaired older adults, neural selectivity plays an important role in associative memory differences that occur with age, diminished top-down attention, and preclinical AD pathology. Second, the SEM revealed that both the AD-related (*a*_1_*d*_21_*b*_2_ = −0.002, β_standardized_ = −0.016, 95% CI = −0.005 to −0.0005) and attention-related (*a*_3_*d*_23_*b*_2_ = −0.001, β_standardized_ = −0.011, 95% CI = −0.004 to −0.0001) pathways explain unique variance in memory performance through unique variance in reduced neural selectivity (Fig. 6A). Corresponding SEMs predicting out-of-task performance (i.e., delayed recall and MST performance) provided further evidence for multiple pathways between age, neural selectivity, and memory (see Supplementary Result).

To further summarize the unique and shared contributions of age, neural selectivity, DAN SME, plasma pTau_181_, sex, and education in predicting place associative *d*′, we computed hierarchical linear models, as outlined in table S11. The results revealed that neural selectivity explained the largest unique variance in place associative *d*′ (*R*^2^_NS_ = *R*^2^_total_ − *R*^2^_1a_ = 4.4%), followed by age (*R*^2^_Age_ = *R*^2^_total_ − *R*^2^_1c_ = 2.8%) and DAN SME (*R*^2^_DAN_ = *R*^2^_total_ − *R*^2^_1b_ = 2.4%) and lastly by plasma pTau_181_ (*R*^2^_AD_ = *R*^2^_total_ − *R*^2^_1d_ = 1.3%) (Fig. 6B). Plasma pTau_181_ shared considerable variance (*R*^2^_sharedAD_ = *R*^2^_3a_ − *R*^2^_AD_ = 7.2%) with other predictors, especially with age (*R*^2^_Age&AD_ = *R*^2^_total_ − *R*^2^_Age_ − *R*^2^_AD_ − *R*^2^_2a_ = 2.1%), in explaining memory (Fig. 6C).

## DISCUSSION

The current study systematically examined mechanisms of within- and across-individual differences in neural selectivity and explored their impact on episodic memory in cognitively unimpaired older adults. Within-individual, neural selectivity in place- and face-selective ROIs and activity in frontoparietal nodes of the DAN were significantly higher when encoding later remembered than later forgotten trials. Moreover, holding memory constant, trial-level neural selectivity for preferred stimuli positively scaled with trial-level DAN activity. Across-individual, neural selectivity significantly decreased with age, principally on later remembered trials, with the decrease reflecting attenuated activation to preferred stimuli. Moreover, higher memory-related DAN activity and lower plasma pTau_181_ levels were associated with greater neural selectivity, principally on subsequently remembered trials in place-selective regions. A path analysis integrated the effects of age, DAN activity, plasma pTau_181_, and neural selectivity on memory performance, uncovering distinct age-related and age-independent pathways from AD pathology and diminished top-down attention network activity to reduced neural selectivity that ultimately predicted reduced episodic memory. Collectively, these findings indicate that the quality of cortical representations during learning is influenced by multiple factors, including age, top-down attention, and AD pathology, with distinct interactions between these factors and neural selectivity having an impact on successful episodic memory encoding and later remembering in cognitively unimpaired older adults.

Age-related changes in neural selectivity are well documented (*10*–*12*, *14*, *66*), with prior studies typically adopting a between-groups approach that revealed lower neural selectivity in older compared to younger adults [for individual-difference data, see Park *et al.* (*65*); compared to Voss *et al.* (*67*)]. Here, we used a combined within- and across-individual difference approach with a large sample of cognitively unimpaired older adults and demonstrated an interaction between age and subsequent memory in neural selectivity. Specifically, neural selectivity during encoding in cognitively unimpaired older adults was greater on subsequently remembered compared to forgotten trials, suggesting that, within individuals, the precision of cortical representations of events during learning is important for later remembering (*12*, *68*) and, moreover, between individuals, this precision on subsequently remembered trials declined with increasing age later in the lifespan. These findings link event-level neural selectivity to event-level later remembering in older adults, suggesting that neural selectivity reflects, in part, the strength or precision of internal (cortical) representations of event features as events unfold and that this precision predicts future remembering when those features are the targets of future retrieval attempts. These findings extend past studies in younger adults that demonstrate that the strength of cortical representations at encoding predicts subsequent memory (*24*, *25*, *69*, *70*), revealing that age-related declines in the precision or strength of cortical representations (*71*) that occur across the later lifespan are differentially observed on subsequently remembered trials. This suggests that even on successfully encoded trials (i.e., events later remembered), memory strength and/or precision continue to decline from one’s 60s to 80s, a possibility that should be explored in future fMRI studies that include quantitative behavioral assays of memory precision (*72*, *73*).

We observed a trend for a greater age-related decline in place-associative versus face-associative memory performance, which was accompanied by evidence that the age-related decline in neural selectivity was greater in scene-selective than in face-selective regions [see also Srokova *et al.* (*74*)]. This decline stemmed from reduced activity in response to preferred stimuli (i.e., scenes) rather than an enhanced response to nonpreferred stimuli (i.e., faces), consistent with neural attenuation (*18*, *65*, *74*). Extant data indicate that age-related declines in neural selectivity are consistently observed in scene-selective areas, whereas evidence for changes in neural selectivity in face- and/or object-selective regions is more variable (*10*, *15*, *18*, *67*, *74*–*76*). One possibility is that faces consist of more typical (i.e., overlapping) features and, when using famous faces (as in the present study), participants may also shift encoding to differentially rely on semantic versus perceptual features. By contrast, the visual representations of scenes (including landmarks, as here) may be more distinctive, providing an opportunity to detect age-related variation in perceptual encoding and neural selectivity (*74*, *77*). Another possibility is the “last in, first out” hypothesis, which posits that brain regions that mature last are the first to degenerate with age (*78*, *79*). While face processing areas appear to mature early (*80*, *81*), regions involved in scene processing, especially the RSC and OPA (*82*), appear to emerge later and may reach maturity as late as early adulthood (*83*–*85*). This delayed maturation may render scene-selective regions more susceptible to factors that drive age-related declines in neural selectivity.

The present focus on factors driving age-related declines in neural selectivity revealed that top-down attention during encoding, indexed indirectly using the DAN SME, (i) predicts subsequent memory (*27*, *29*, *64*, *86*), (ii) modulates neural selectivity (*20*, *23*, *26*), (iii) does not vary with plasma or CSF biomarkers of preclinical AD, and (iv) partially accounts for age-independent and age-related variability in memory through its impact on neural selectivity. Controlling for age, cognitively unimpaired older adults with greater memory-related neural activity in the DAN demonstrated higher neural selectivity during subsequently remembered events, with the DAN SME being the top predictor of variance in neural selectivity. Moreover, trial-by-trial fluctuations in DAN activity (restricted to in-scanner subsequent associative hit trials) predicted trial-wise neural selectivity within participants. This finding complements the relationship between DAN SME and neural selectivity and suggests that this relationship reflects, at least in part, the influence of top-down attention on neural selectivity. Hence, greater top-down attention, supported by DAN (*35*, *53*, *55*, *62*), may enhance the precision of perceptual representations in visual cortex (*24*, *26*) with impacts on later memory. Notably, the age-independent association between neural selectivity and DAN activity was significantly stronger compared to neural selectivity’s relationship with VAN activity, indicating that neural selectivity during successful encoding is predominantly modulated by top-down rather than bottom-up attention network activation (*53*, *62*, *87*, *88*). SEM also suggested that age-related differences in memory stem, in part, from age-related change in DAN activity, which leads to declines in neural selectivity.

Potentially in line with our findings, prefrontal dopamine D1 receptor–mediated activity enhances the magnitude and orientation selectivity of neural responses in the visual cortex of macaques (*89*); prefrontal dopamine plays a central role in the modulation of top-down attention (*90*, *91*); and, with age, the densities of dopamine D1/D2, serotonin, and acetylcholine receptors in human frontal cortex markedly decrease, contributing to age-related cognitive dysfunction (*92*). Aging is also associated with structural (*57*–*59*) and functional (*60*, *61*) changes in and between frontoparietal nodes of the DAN, which likely contribute to disruption of top-down attention. Thus, attention-mediated age-related decline in neural selectivity may result, in part, from dysregulated frontoparietal neurotransmission—possibly stemming from receptor, neurotransmitter, and connectivity changes—that disrupts top-down attentional modulation of perceptual representations of event features. The present observation that reduced neural selectivity reflects attenuated activation to preferred stimuli is compatible with diminished top-down gain modulation.

While early AD pathology (e.g., plasma pTau_181_) was unrelated to memory-related engagement of top-down attention network activation, our data reveal that early AD pathology was associated with reduced neural selectivity and partially accounted for age-related memory decline via neural selectivity. Moreover, we observed an age-independent relationship between early AD pathology and neural selectivity. Consistent with previous tau PET findings (*47*), neural selectivity’s negative relationship with early AD pathology was principally observed in place-selective regions. However, whereas Maass *et al.* (*47*) observed evidence for functional response broadening (i.e., increased activation to nonpreferred categories), the present findings are ambiguous with respect to whether neural selectivity change with early AD pathology reflects attenuation and/or broadening (Fig. 3F). Given that our study only examined global biofluid measures of AD (plasma and CSF measures of pTau_181_), we are unable to address whether differences in neural selectivity relate to focal tau within the medial temporal lobe and/or cortical regions important for category-specific effects.

The present data revealed that neural selectivity at category level accounted for age-independent variability in memory, not only within-task (i.e., in-scanner associative memory *d*′ and postscan cued recall performance) (*15*, *18*, *74*) but also across-task (i.e., mnemonic discrimination and delayed recall composite performance measured weeks apart from the fMRI session). In contrast, neural selectivity did not vary with a composite executive function measure that spanned processing speed (*18*), working memory, and verbal fluency (*74*). Evidence that category-level neural selectivity at encoding predicts performance on multiple memory measures acquired in different temporal and spatial contexts implies that individual differences in selectivity relate, in part, to stable differences in memory abilities (i.e., “trait”-like), rather than solely to transient state differences between people at the time of measurement. This observation should motivate future research to explore whether neural selectivity predicts longitudinal memory decline and progression to mild cognitive impairment.

Related to the latter, given the cross-sectional nature of the present study, it is difficult to establish causality and temporal relationships between neural selectivity and other variables. An ongoing longitudinal study using a 7-year follow-up with the present sample will ultimately help address these questions. We also note that, although the present sample size of deeply phenotyped older adults is relatively large, it lacks educational and racial/ethnic diversity that potentially limits the generalizability of the findings. Future studies should include more representative samples, which is also one of our future directions. In addition, as mentioned previously, this study did not include a direct measure of top-down attention. Given the well-established role of the DAN in supporting top-down attentional control (*35*, *53*, *55*, *62*), the DAN SME was used as an indirect neural index of top-down attention. This approach revealed that higher DAN activity, both at the participant level and trial level, was associated with greater neural selectivity, suggesting that top-down attention may contribute, at least in part, to neural selectivity. While the DAN is primarily implicated in top-down attention, it also is posited to support other cognitive functions, such as visuospatial working memory (*93*) and task switching (*94*). Therefore, the observed relationship between DAN activity and neural selectivity may not be solely attributable to top-down attention. For instance, reductions in univariate activity for remembered preferred stimuli might also reflect predictive coding mechanisms (*95*) or increased neural efficiency associated with successful memory encoding (*96*). Future studies could further investigate these alternative explanations by incorporating experimental manipulations that disentangle top-down attention from other cognitive processes. Furthermore, in our study, the intraparietal sulcus (IPS) and frontal eye field (FEF) nodes of the DAN were defined on the basis of the resting-state functional connectivity literature. While prior research has demonstrated that resting-state parcellation aligns well with task-based functional localization (*97*), future studies could target the attentional specificity of DAN regional definition using functional localizers using top-down attention–related tasks, such as selective attention paradigms (*30*). Last, while we observed that early AD biomarkers relate to neural selectivity and memory performance, but not to top-down attention, the variance explained by these biomarkers was modest, especially in the subsample of participants with available CSF. This likely reflects our focus on cognitively unimpaired older adults, who are, by definition, characterized as demonstrating cognition (including memory) in the normal range, along with the restricted number of participants with elevated AD biomarkers. PET assays of regional tau burden may ultimately provide sensitive and complementary insights into how AD pathology affects neural selectivity and, in doing so, memory.

In sum, this study revealed age-related and age-independent factors that influence cortical representations during event encoding and predict subsequent memory performance. Integrating age, early AD pathology, top-down attention network activation, and neural selectivity, we elucidated multiple pathways underlying individual differences in episodic memory among cognitively unimpaired older adults. On the one hand, controlling for age, AD-independent reductions in top-down attention and AD-related pathology alter the precision of cortical representations of event features during experience, with consequences for future remembering. On the other hand, age-related episodic memory decline is partially accounted for by reduced neural selectivity, that is, in turn, independently affected by top-down attention and early AD pathology. Hence, these data inform models of the factors—related to and independent of age and early AD processes—that affect memory formation and account for why some cognitively unimpaired older adults remember better than others.

## MATERIALS AND METHODS

### Participants

This study includes data from 166 cognitively unimpaired older adults (60 to 88 years, 95 female; Table 1) of an initial 212 participants (60 to 88 years, 121 female) enrolled in the SAMS (*6*, *36*). SAMS eligibility included normal or corrected-to-normal vision and hearing, right-handed, native English speaking, no history of neurological or psychiatric disease, a Clinical Dementia Rating score of zero (*98*), and performance within the normal range on a neuropsychological assessment. Inclusion in the current analyses additionally required the availability of both structural MRI and task-based fMRI data, which were collected from 170 participants. Among these, four participants were excluded from all analyses due to excess head motion during scanning (*n* = 3; see MRI data acquisition and preprocessing) or visible artifacts in the fMRI data (*n* = 1). This resulted in available data from 166 older adults that were included in group-level univariate generalized linear model (GLM) analyses. Of the 166 participants with an acceptable quality of fMRI data, 10 participants were excluded from the individual-difference analyses due to having fewer than three trials under at least one of the four conditions [category (i.e., face versus place) × memory (i.e., remembered versus forgotten)]. Thus, 156 participants are included in the individual-difference analyses (Table 1). All participants provided informed consent in accordance with a protocol approved by the Stanford Institutional Review Board.

**Table 1.** Participant demographics and biomarker summary. ID, individual difference. Values are *n* (%) or means ± SD. Categorical amyloid status was defined by either CSF Aβ_42_/Aβ_40_ [cutoff of 0.0752 based on Trelle *et al.* (*6*)] or Aβ-PET Centiloid unit (cutoff of 18). Seventeen participants with plasma did not have corresponding CSF or PET assays to compute amyloid status.

		Full fMRI-encoding sample (*n* = 166)	fMRI-encoding ID subsample (*n* = 156)	fMRI-encoding ID-plasma subsample (*n* = 138)	fMRI-encoding ID-CSF subsample (*n* = 115)
Age, year		68.95 ± 5.71	68.77 ± 5.68	68.86 ± 5.80	68.29 ± 5.35
Education, year		16.69 ± 2.03	16.77 ± 2.01	16.83 ± 2.07	16.77 ± 2.17
Female		95 (57.23)	89 (57.05)	81 (58.70)	68 (59.13)
pTau_181_, pg/ml		–	–	1.59 ± 0.54 (*n* = 137)	39.02 ± 22.14
Aβ_42_/Aβ_40_		–	–	0.10 ± 0.01 (*n* = 136)	0.09 ± 0.02
Apolipoprotein E-e4	Carries	42 (25.30)	40 (25.64)	36 (26.09)	28 (24.35)
	Noncarries	122 (73.49)	114 (73.08)	102 (73.91)	86 (74.78)
	Missing	2 (1.20)	2 (1.28)	–	1 (0.87)
Amyloid status	Positive	35 (21.08)	32 (20.51)	31 (22.46)	29 (25.22)
	Negative	106 (63.86)	102 (65.38)	90 (65.22)	86 (74.78)
	Missing	25 (15.06)	22 (14.10)	17 (12.32)	–

### Biofluid data

#### Plasma data

Plasma data were available from 138 participants who completed a blood draw within 21.14 weeks (means ± SD = 5.33 ± 3.31 weeks) of the fMRI session. EDTA plasma was collected by venipuncture, centrifuged for 10 min at 2000*g* at 22°C, aliquoted in polypropylene tubes, and stored at −80°C until measurement. Using procedures previously described (*99*), plasma Aβ_42_ and Aβ_40_ (*n* = 136) and pTau_181_ (*n* = 137) were measured using the fully automated LUMIPULSE G1200 instrument (Fujirebio Inc., Malvern, PA). A single aliquot for each participant was used to measure Aβ_42_ and Aβ_40_ in a single batch analysis, and a single aliquot was used to measure pTau_181_ in a separate single batch analysis. Analyses were conducted by the Stanford Alzheimer’s Disease Research Center (ADRC) Biomarker Core.

#### CSF data

CSF data were available from 115 participants who completed a CSF draw within 19 weeks (means ± SD = 5.14 ± 3.83 weeks) of the fMRI session. As described previously (*6*), CSF samples were centrifuged for 15 min at 500*g* at 22°C, aliquoted in polypropylene tubes, and stored at −80°C until measurement. Using procedures previously described (*100*), a single aliquot for each participant was used to measure Aβ_42_, Aβ_40_, pTau_181_, and total tau using the fully automated LUMIPULSE G1200 instrument in a single-batch analysis by the Stanford ADRC Biomarker Core.

### fMRI task

#### Word-picture associative memory task

The word-picture associative memory task (Fig. 1A) was administered concurrent with fMRI as previously described (*6*, *36*). Briefly, participants first encoded word-face and word-place associations and then engaged in an associative retrieval task. The word-picture pairs comprised concrete nouns (e.g., “banana” and “violin”) paired with pictures of famous faces (e.g., “Queen Elizabeth” and “Ronald Reagan”) or well-known places (e.g., “Golden Gate Bridge” and “Niagara Falls”). The task consisted of five alternating study and test blocks. Each study block included 12 word-face and 12 word-place pairs; participants were instructed to form a link between the word and picture presented. In each test block, participants saw a mix of 24 studied words and 6 novel (foil) words. Memory was assessed using an associative cued recall test accompanied by a button response: Participants selected “face” or “place” if they remembered the word and could recall the associated picture or picture category; they selected old if they remembered the word but could not recall the associated picture or picture category; they selected new if they did not remember studying the word. Associative memory performance was estimated using a sensitivity index, associative *d′*, where hits were defined as correct associative category responses to studied words and false alarms were defined as incorrect category responses to new words. Thus, associative *d′* = *Z*(“correct associate category”|old) − *Z*(“associate category”|new). Similar calculations were performed for place [*Z*(“place correct associate category”|old place-word pairs) − *Z*(“place associate category”|new)] and face [*Z*(“face correct associate category”|old face-word pairs) − *Z*(“face associate category”|new)] associative *d*′, respectively. Notably, trials with no responses were excluded when calculating associative *d′*.

Following the whole MRI scanning session (~2 hours), participants completed a cued recall test in a different room outside the scanner to assess their ability to recall the specific face or place associated with each cue word (i.e., exemplar-specific retrieval). During this posttest, all studied words were presented in a randomized sequence, and participants were asked to recall the name of the associated image. If they could not remember the name, then they were encouraged to describe the associated image in as much detail as possible. The test was self-paced, with responses typed out on a keyboard. If participants were unable to recall any details about the associated image, then they were instructed to leave the response blank.

The typed responses were initially processed using custom R scripts to identify exact matches to the names of the studied images. Responses that did not match exactly were flagged and subsequently evaluated by a human rater, who determined the degree of correspondence between the participant’s description and the correct associated image. The proportion of studied words for which the associated specific exemplar was correctly recalled (*N*_exemplar correct_/*N*_all old_) was calculated. Of 156 participants who passed fMRI quality control and were included in individual difference analyses, 6 did not complete the posttest, resulting in 150 participants being included in the analysis of the posttest data.

### MRI data acquisition and preprocessing

Data were acquired on a 3-T GE Discovery MR750 MRI scanner (GE Healthcare) using a 32-channel radiofrequency receive-only head coil (Nova Medical). Functional data were acquired using a multiband echo planar imaging (EPI) sequence (acceleration factor = 3) consisting of 63 oblique axial slices parallel to the long axis of the hippocampus [repetition time (TR) = 2 s, echo time (TE) = 30 ms, field of view (FOV) = 215 mm by 215 mm, flip angle = 74°, and voxel size = 1.8 mm by 1.8 mm by 2 mm]. To correct for main static magnetic field (B_0_) distortions, we collected two B_0_ field maps before every functional run, one in each phase encoding direction. Structural MRI included a whole-brain high-resolution T1-weighted anatomical volume (TR = 7.26 ms, FOV = 230 mm by 230 mm, voxel size = 0.9 mm by 0.9 mm by 0.9 mm, and slices = 186).

MR data were preprocessed using fMRIPrep 23.0.0rc0 (*101*) (RRID: SCR_016216), which is based on Nipype 1.8.5 (*102*, *103*) (RRID: SCR_002502). Functional images were corrected for susceptibility distortion, head motion, and slice timing and coregistered to the T1-weighted structural volume. For the group-level analysis, we resampled BOLD runs into standard volumetric (i.e., ICBM 152 Nonlinear Asymmetrical template version 2009c) and surface (i.e., fsaverage) spaces. Grayordinates files (*104*) containing 91,000 samples were generated using the highest-resolution fsaverage as intermediate standardized surface space.

Images with motion artifacts were automatically identified as those TRs in which total displacement relative to the previous frame exceeded 0.9 mm, a threshold commonly used in aging studies (*105*–*107*) and corresponding to approximately half a voxel in size. Trials with frame-wise displacement (FD) of any time points exceeding 0.9 mm were identified as artifacts. Runs in which the number of artifacts identified exceeded 25% of TRs or 50% of trials, as well as runs in which FD exceeded 5 mm, were excluded. These criteria led to exclusion of data from three participants who exhibited excess head motion across runs, as well as exclusion of a varying number of studies and test runs from five additional participants. Both encoding and retrieval fMRI data were preprocessed, but we report only on encoding data in the current study [for results at retrieval, see Trelle *et al.* (*36*)].

### Whole-brain univariate analysis

Before conducting univariate activation analyses, surface-based and volume-based data were spatially smoothed using a 6-mm full width at half maximum Gaussian kernel and filtered in the temporal domain using a nonlinear high-pass filter with a 100-s cutoff. Specifically, the wb_command was used for surface-based smoothing, and SUSAN was used for volume-based smoothing. Univariate activation analysis in volumetric space was performed for a leave-one-participant-out procedure (see the “Definition of ROIs” section and Fig. 2) and ROI activity extraction (see the “ROI-based univariate analysis” section). A GLM within the FMRIB’s Improved Linear Model (FILM) module of FMRIB Software Library (FSL) was used to model the data. During encoding, word-face and word-place pairs were conditioned on subsequent memory, modeling subsequent associative hits, associative misses, item hits, item misses, and no responses separately. Events were modeled as boxcar functions based on the time of stimulus onset and duration (4 s) and convolved with the canonical hemodynamic response function (double gamma function). The category effect was defined as the difference between word-face and word-place pairs, and the SME was defined as the difference between subsequently remembered associations (i.e., associative hits) and forgotten associations (i.e., associative misses, item hits, and item misses) in the scanner. These contrasts were then submitted to group-level analyses using FSL Permutation Analysis of Linear Models (https://fsl.fmrib.ox.ac.uk/fsl/fslwiki/PALM) with 1000 iterations and tail acceleration (*108*). Unless otherwise noted, significant results are reported after whole-brain correction for multiple comparisons, using a two-step approach: first, controlling for family-wise error (FWE) within each of the left hemisphere, right hemisphere, and subcortical regions with *P* < 0.05 using threshold-free cluster enhancement (TFCE) (*109*) and, second, using Bonferroni correction for the three macroscopic regions [i.e., left hemisphere, right hemispheres, and subcortical; i.e., −log_10_(α/*N*) = −log_10_(0.05/3) = 1.7782].

### Single-trial neural activity

A GLM was used to estimate the activation pattern for each trial of the word-picture pairs during encoding, computed in native space (i.e., T1 space). The same preprocessing procedure as in the univariate analysis was applied, except that no spatial smoothing was performed. A least squares single method was used in this single-trial model, where the target trial was modeled as one explanatory variable (EV) and all other trials were modeled as another EV (*110*). Each trial was modeled at its presentation time and convolved with a canonical hemodynamic response function (double gamma). This voxelwise GLM was used to compute the activation associated with each trial in the task. The resulting activation brain map per trial, represented as a *t*-statistical map (*111*), was then used to calculate a multivariate index of neural selectivity through PSA.

### Definition of ROIs

The primary neural selectivity analyses were conducted in face- and place-selective cortical ROIs defined in a two-step process. First, a priori ROIs were selected on the basis of an independent probabilistic functional atlas that shows the likelihood of a given voxel being located in the face- or place-selective regions (probability > 0.1) (*112*), with the OFA and FFA defined as face-selective regions and the OPA, PPA, and RSC defined as place-selective regions (Fig. 1D). Second, these masks were intersected with whole-brain univariate category effect masks from the current experiment’s encoding phase data. To ensure statistical independence, we used a leave-one-participant-out procedure to define ROIs for each held-out participant (Fig. 2). Specifically, for a given participant, we performed group-level analysis excluding this participant and obtained a whole-brain category effect map in Montreal Neurological Institute (MNI) space, after controlling for FWE with *P* < 0.05 through TFCE (*109*). We then intersected the participant-independent whole-brain category map with the predefined functional ROIs (*112*), and each resulting ROI mask was transformed to the participant’s native space using antsApplyTransforms and defined as all voxels intersecting the midpoint between the gray-white and gray-pial boundaries. For the OFA ROI, 10 participants were excluded from the analysis (number of voxels < 30) due to having only partial coverage of early visual cortex in their FOV.

We additionally examined the role of attention in modulating neural selectivity during encoding. To address this question, we used the Schaefer 2018 atlas (7 networks with 400 parcels) (*113*) to define frontoparietal nodes of the DAN, which included the FEFs and IPS, and the VAN, which included the temporoparietal junction (TPJ) and the inferior frontal cortex (IFC) (Fig. 1E). Specifically, FEF contained all FEF nodes within the DAN (ROI labels: 86 to 89 for left and 290 to 292 for right), IPS contained parietal nodes within the DAN (ROI labels: 74 to 85 for left and 276 to 289 for right), TPJ contained temporal occipital and temporal parietal nodes within the VAN (ROI labels: 92 to 95 for left and 296 to 300 for right), and IFC contained frontal-operculum-insula nodes within the VAN (ROI labels: 97 to 105 for left and 302 to 309 for right). The DAN and VAN ROIs were transformed to the participant’s native space and intersected with the midpoint between the gray-white and gray-pial boundaries.

### ROI-based univariate analysis

All ROI-based analyses were conducted in the participants’ native space (i.e., T1 space). To examine neural selectivity in each face- and place-preferred region, we performed a whole-brain participant-level univariate analysis in native space without spatial smoothing and extracted the mean contrast estimate (*t* value) of face versus place trials from the ROI for each subsequent in-scanner memory condition (e.g., remembered: associative hit; forgotten: associative miss, item hit, and item miss). Neural selectivity in face-preferred regions (i.e., FFA and OFA) was defined as face-place and vice versa for place-preferred regions (i.e., PPA, OPA, and RSC). Similarly, we extracted face- and place-related activity (relative to the implicit baseline) in each face- and place-preferred ROI. The univariate SME in the DAN (i.e., mean *t* value in FEF and IPS) and VAN (i.e., mean in TPJ and IFC) was extracted from the contrast of remembered trials (i.e., associative hit trials) versus Forg trials (i.e., associative miss, item hit, and item miss trials).

### ROI-based PSA

To estimate trial-level neural selectivity within face- and place-preferred regions, we applied PSA using single-trial activation estimates. For each category-specific ROI and trial, we first computed within-category similarity by measuring the pattern similarity between the given trial and all other trials of the same category. Next, we calculated across-category similarity by measuring the pattern similarity between the given trial and all trials from the other category. To reduce confounds from autocorrelation and subsequent memory, we excluded within-run pairs and only included associative hit trials in both the within- and across-category similarity calculations. In addition, mean activation for each pair was regressed out from the pattern similarity after Fisher’s *z*-transformation to minimize its influence on neural similarity (*114*). The resulting residualized neural similarity measures were used to quantify trial-level neural selectivity, computed as the difference between the mean within-category and mean across-category similarities (*15*, *115*).

### Out-of-scanner independent cognitive tasks

#### Standardized cognitive composite scores

Test scores from a neuropsychological battery completed by all participants during study enrollment (*36*) were used to create two composite cognitive scores: a delayed recall score and an executive function score. The delayed recall score (*6*, *36*) was composed of performance across (i) the logical memory subtest of the Wechsler Memory Scale, (ii) the Hopkins Verbal Learning Test–Revised, and (iii) the Brief Visuospatial Memory Test–Revised. The executive function score was composed of performance across tests measuring (i) task switching (Trail Making Test B, inverse), (ii) working memory (WMS-III digit span total score), and (iii) category fluency (animal naming). Composite scores were computed by first *z*-scoring individual subtest scores using the full SAMS sample as a reference and then averaging. These neuropsychological data were collected 21.14 weeks (means ± SD = 4.94 ± 3.69 weeks) from the fMRI session.

#### Mnemonic similarity task

The MST was administered using previously described measures and instructions (*6*, *116*) and was collected within 15.86 weeks (means ± SD = 4.79 ± 3.45 weeks) of the fMRI session. During an incidental encoding phase, participants made indoor/outdoor judgments for 128 pictures of everyday objects. Participants then performed a surprise memory test, in which 64 of the studied objects were intermixed with 64 perceptually similar lure objects and 64 novel (dissimilar) objects. Participants were to respond old if they remembered the object as having been studied, “similar” if they remembered the object as similar, but not identical, to a studied object, or new if they remembered the object as not having been studied. The 64 lures systematically varied in perceptual similarity to the untested studied objects across five levels of similarity/difficulty. Here, the performance was estimated for each level of target-lure similarity using two sensitivity indices: (i) lure/new *d*′, the ability to correctly classify perceptually similar lures and differentiate them from novel objects, as *Z*(similar|lure) − *Z*(similar|novel foil); and (ii) old/lure *d′*, the ability to correctly endorse studied objects and avoid the propensity to incorrectly endorse lures as old, as *Z*(old|target) − *Z*(old|lure). Building on prior work (*6*), the primary measure of interest was the degree to which performance improved as a function of decreasing target-lure similarity (i.e., decreasing task difficulty). LMMs were used to model performance across the five similarity levels and to examine interactions with measures of neural selectivity. LMMs included a random intercept and slope for each participant. Participant-specific slopes, which reflected the magnitude of the increase in performance as target-lure similarity moved from high to low (i.e., decreasing difficult), were extracted and plotted against neural selectivity to visualize similarity × selectivity interactions. MST data were available from 135 participants.

### Statistical analysis

#### Linear mixed-effect model

For repeated-measures models exploring effects of category (face and place), subsequent memory (remembered and forgotten), and region (face-selective and place-selective) on neural selectivity, LMMs were used to examine the relationship between dependent and independent variables with lmer function from the lmerTest package (https://cran.r-project.org/web/packages/lmerTest/index.html) in R 4.3.0 (R Core Team 2023). Participants were set as random effects. The degrees of freedom in the mixed models were estimated by the Satterthwaite approximation. Unless otherwise specified, *P* values were corrected using Holm-Bonferroni correction for multiple comparisons (termed as *P*_Holm_).

#### Direct comparisons for two correlation coefficients

To examine whether neural selectivity on subsequently remembered trials was more strongly related to memory performance than on forgotten trials, we conducted direct comparison analyses using the R package “cocor” (https://cran.r-project.org/web/packages/cocor/index.html), which provides a comprehensive solution to compare two correlations based on dependent groups with overlapping variables (i.e., associative *d*′). We report statistical results based on the computation of CIs (*117*) since these tests are generally considered superior to significance tests because they indicate, respectively, the magnitude and precision of the estimated effect.

#### Mediation analysis

Mediation effect tests were implemented with R package lavaan (https://cran.r-project.org/web/packages/lavaan/index.html). For simple mediation analyses, we examined the relationship between (i) mediators (*M*) and independent variables (*X*) (*M* = *k*_1_ + *aX* + ε_1_) and (ii) independent variables (*X*) and dependent variables (*Y*) with mediator (*M*) (*Y* = *k*_2_ + *c*′X + *bM* + ε_2_). Sex and years of education were included in models as confounding variables. We also included age as a covariate when age was not a variable of interest in the model. The indirect effect was estimated as *ab*, and the direct effect was estimated as *c*′. The direct and indirect effects sum to the total effect, *ab + c*′.

A multiple mediation analysis was used to examine the mediation effects of early AD pathology (plasma pTau_181_) and top-down attention (DAN SME) for the relationship between age and neural selectivity. Specifically, we examined the relationships between (i) plasma pTau_181_ (M1) and age (*X*) (M1 = *k*_1_ + *a*_1_*X* + ε_1_); (ii) top-down attention (M2) and age (*X*) (M2 = *a*_2_X + ε_2_); and (iii) neural selectivity (*Y*) with age (*X*), plasma pTau_181_ (M1), and top-down attention (M2) (*Y* = *k*_2_ + *c*′*X* + *b*_1_M1 + *b*_2_M2 + ε_3_). Sex and years of education were included in the model as confounding variables. In the above equations, *X* (age) is the predictor, *Y* (neural selectivity in place-selective region on later remembered trials) is the dependent variable, and M1 (plasma pTau_181_) and M2 (DAN SME) are the mediators.

Unless otherwise specified, the significance of indirect effects was determined by 95% CI based on 5000 bootstrap resampling. In addition, to ensure comparability of β values across different pathways, we also report standardized β estimates using a completely standardized solution (i.e., std.all) in the parameterEstimates function provided by lavaan. The same approaches were used for the SEM below.

#### Structural equation modeling

The lavaan was used to conduct SEM to examine whether individual differences in episodic memory in cognitively unimpaired older adults were affected by two independent pathways (Fig. 6A): (i) age ➔ plasma pTau_181_ ➔ neural selectivity (restricted to place-selective regions on later remembered trials, hereafter) ➔ memory performance and (ii) age ➔ top-down attention ➔ neural selectivity ➔ memory performance. To do this, we tested the relationships between (i) plasma pTau_181_ (M1) with age (*X*) (M1 = *k*_1_ + *a*_1_*X* + ε_1_); (ii) neural selectivity (M2) with age (*X*), top-down attention (M3; i.e., DAN SME), and plasma pTau_181_ (M1) (M2 = *k*_2_ + *a*_2_*X* + *d*_21_M1 + *d*_23_M3 + ε_2_); (iii) top-down attention (M3) with age (*X*) (M3 = *a*_3_X + ε_3_); and (iv) memory performance (*Y*) with age (*X*), plasma pTau_181_ (M1), neural selectivity (M2), and top-down attention (M3) (*Y* = *k*_3_ + *c*′*X* + *b*_1_M1 + *b*_2_M2 + *b*_3_M3 + ε_4_). Sex and years of education were included to the model as confounding variables. In the above equations, *X* (age) is the predictor, *Y* (memory performance, e.g., associative *d*′, delayed recall composite score, and MST old/lure *d*′ similarity slope) is the dependent variable, and M1 (plasma pTau_181_), M2 (neural selectivity in place-selective region on later remembered trials), and M3 (DAN SME) are the mediators. The indirect effect of age ➔ plasma pTau_181_➔ neural selectivity ➔ memory performance was estimated as *a*_1_ × *d*_21_ × *b*_2_, and the other indirect effect of age ➔ top-down attention ➔ neural selectivity ➔ memory performance was estimated as *a*_3_ × *d*_23_ × *b*_2_. The total effect (*c*) equals *c*′ *+ a*_1_*d*_21_*b*_2_ *+ a*_1_*b*_1_ *+ a*_2_*b*_2_ *+ a*_3_*b_3_ + a*_3_*d*_23_*b*_2_.

## Supplementary Materials

### This PDF file includes:

Supplementary Text Tables S1 to S20 Figs. S1 to S9 References
