# Supplementary material for: Top-down attention and Alzheimer’s pathology affect cortical selectivity during learning, influencing episodic memory in older adults
Source: Sci Adv. 2025 Jun 13;11(24):eads4206. doi: 10.1126/sciadv.ads4206 (PMC12164959; doi:10.1126/sciadv.ads4206)
Supplement: Supplementary file 1 — Supplementary Text Tables S1 to S20 Figs. S1 to S9 References [file sciadv.ads4206_sm.pdf]

Science Advances
AAAS

# Supplementary Materials for

## Top-down attention and Alzheimer's pathology affect cortical selectivity during learning, influencing episodic memory in older adults

Jintao Sheng *et al.*

Corresponding author: Jintao Sheng, jsheng4@stanford.edu; Anthony D. Wagner, awagner@stanford.edu

*Sci. Adv.* **11**, eads4206 (2025)
DOI: 10.1126/sciadv.ads4206

**This PDF file includes:**

Supplementary Text
Tables S1 to S20
Figs. S1 to S9
References

**Supplementary Text**
**Supplementary Results**

**Relationship between age and AD biomarkers**

Age was associated with increased log-transformed $pTau_{181}$ (plasma: $\beta = .009$, $p < .001$; CSF: $\beta = .013$, $p < .001$) and decreased $A\beta_{42}/A\beta_{40}$ ratio (plasma: $\beta = -.0005$, $p = .026$; CSF: $\beta = -.001$, $p = .006$). The plasma and CSF assays were correlated ($pTau_{181}$: $R = .37$, $p < .001$, n = 104; $A\beta_{42}/A\beta_{40}$: $R = .51$, $p < .001$, n = 103; **Fig. S7**).

**Relationship between bottom-up attention and neural selectivity**

To examine if attention effects on neural selectivity are specific to DAN-mediated top-down attention or reflect more general impacts of attentional processes on selectivity, we conducted parallel analyses with frontoparietal regions of the ventral attention network (VAN; **Fig. 1E**) linked to bottom-up attentional processes (*53*). While VAN activity demonstrated a significant SME ($t_{155} = 3.98$, $p < .001$), its magnitude did not significantly vary with age ($\beta = .001$, $p = .880$). A linear mixed-effect model (LMM), with bottom-up attention (VAN SME), memory (Rem vs. Forg), and region (Face- vs. Place-selective ROI) as predictors of neural selectivity, revealed a three-way interaction ($F_{1,462} = 4.276$, $p = .039$), but the main effect of bottom-up attention was not significant ($F_{1,151} = 1.608$, $p = .207$). Moreover, the simple effect between VAN SME and neural selectivity was not significant in place- (Rem: $\beta = .302$, $p_{Holm} = .124$; Forg: $\beta = -.158$, $p_{Holm} = .603$) nor face-selective regions (Rem: $\beta = .114$, $p_{Holm} = .603$; Forg: $\beta = .179$, $p_{Holm} = .603$) (**Fig. S3B**). Critically, a direct comparison revealed that the association between DAN SME and neural selectivity on remembered trials was significantly stronger than that observed with VAN SME (Place-selective ROI: $\Delta r_{DAN\text{-}VAN} = .188$, 95% CI = [.084, .292]; Face-selective ROI: $\Delta r_{DAN\text{-}VAN} = .141$, 95% CI = [.034, .245]). These results provide evidence for a stronger role of top-down, relative to bottom-up, attention in modulating neural selectivity in category-selective cortex during memory encoding in CU older adults.

**Neural selectivity in place-selective regions is associated with face and place associative *d'***

In place-selective regions, we found that neural selectivity related to both place (Rem: $\beta = .366$, $p_{Holm} < .001$; Forg: $\beta = -.323$, $p_{Holm} = .053$; $\Delta r_{Rem\text{-}Forg} = 0.503$, 95% CI = [.337, .656]) and face associative *d'* (Rem: $\beta = .233$, $p_{Holm} = .023$; Forg: $\beta = -.203$, $p_{Holm} = .215$; $\Delta r_{Rem\text{-}Forg} = 0.345$, 95%

CI = [.174, .507]), with LMMs revealing a significant interaction between memory category (i.e., place vs. face associative *d'*) and neural selectivity on later remembered trials ($F_{1,154}$ = 5.616, $p$ = .019), but not on forgotten trials ($F_{1,154}$ = .416, $p$ = .520). This later result suggests that neural selectivity at encoding in place-selective regions differentially contributes to subsequent place associative *d'* than face associative *d'*.

**Neural selectivity in place-selective regions is associated with mnemonic similarity task performance**

Neural selectivity in place-selective regions also explained variance in independent (i.e., out-of-task) memory measures collected during a separate study session separated by weeks from the fMRI sessions. Specifically, neural selectivity on remembered trials related to lure discrimination in the mnemonic similarity task (MST), with a significant similarity × selectivity interaction (**Fig. S8**; lure/new *d'*: $F_{1,130}$ = 5.570, $p$ = .020; old/lure *d'*: $F_{1,130}$ = 9.711, $p$ = .002) revealing higher selectivity was associated with better MST performance at lower-levels of target-lure similarity (**Fig. S8**); that is, there were larger increases in discrimination performance with decreasing levels of target-lure similarity in individuals with higher neural selectivity relative to those with lower neural selectivity (**Fig. 5D**).

**Relationship between neural selectivity in face-selective regions and memory performance**

In face-selective regions, overall associative *d'* did not significantly relate to neural selectivity on remembered trials ($\beta$ = .093, $p_{\text{Holm}}$ = .367), but negatively related to neural selectivity on forgotten trials ($\beta$ = -.319, $p_{\text{Holm}}$ = .026) (**Fig. S9**), and their difference was significant ($\Delta r_{\text{Rem-Forg}}$ = 0.293, 95% CI = [.133, .445]). Although the relationship for subsequently remembered trials was not significant in face-selective regions, it did not statistically differ from that in place-selective regions ($\Delta r_{\text{Face-Place}}$ = -.136, 95% CI = [-.339, .071]); a similar result was observed for forgotten trials ($\Delta r_{\text{Face-Place}}$ = -.092, 95% CI = [-.320, .139]). Similar results were observed when focusing on face associative *d'* (Rem: $\beta$ = .086, $p_{\text{Holm}}$ = .342; Forg: $\beta$ = -.338, $p_{\text{Holm}}$ = .004; $\Delta r_{\text{Rem-Forg}}$ = 0.341, 95% CI = [.183, .492]) or place associative *d'* (Rem: $\beta$ = .045, $p_{\text{Holm}}$ = .647; Forg: $\beta$ = -.254, $p_{\text{Holm}}$ = .053; $\Delta r_{\text{Rem-Forg}}$ = 0.220, 95% CI = [.060, .378]). Mixed-effects models showed that the interaction between memory category (i.e., place vs face associative *d'*) and neural selectivity in face-selective regions was neither significant on remembered trials ($F_{1,154}$ = .034, $p$ = .853) nor on

forgotten trials ($F_{1,154}$ = .534, $p$ = .466). These results suggest that neural selectivity in face-selective regions was not related to memory performance on remembered trials, but negatively related to both face and place associative *d'* on forgotten trials.

**SEMs for overall associative *d'* and face associative *d'***

In addition to predictors of place associative *d'* (reported in the main text), when SEMs were constructed using overall and face associative *d'*, none of the memory-related pathways were significant (**Table S12**). Nonetheless, with the full sample (n = 156), we observed significant mediation effects from age → neural selectivity → associative memory (**Table S13**) and DAN SME → neural selectivity → associative memory (**Table S14**).

**SEMs for out-of-task performance**

We also generated SEMs that explain out-of-task memory performance (delayed recall composite score, n = 137; MST lure/new *d'* and old/lure *d'* similarity slopes, n = 119). The SEMs revealed a significant AD-related pathway ($a_1d_{21}b_2$ = -.0015, 95% CI = [-.004, -.0001]), but nonsignificant attention-related pathway ($a_3d_{23}b_2$ = -.001, 95% CI = [-.003, .0000]), for the delayed recall composite score (**Table S15**). When individual mediation models were performed with the full sample (n = 156), the effects from age → neural selectivity → delayed recall composite score (**Table S16**) and DAN SME → neural selectivity → delayed recall composite score were both significant (**Table S17**). The SEM explaining MST performance (n = 119) revealed that neither the AD- ($a_1d_{21}b_2$) nor attention-related ($a_3d_{23}b_2$) pathway was significant, whereas the pathway from DAN SME → neural selectivity → similarity slope of lure/new *d'* ($d_{23}b_2$) was significant (**Table S18**). Similarly, mediation models with a slightly larger subsample (n = 135) showed neural selectivity significantly mediated the relationship between age and MST performance (**Table S19**) and between top-down attention and MST performance (**Table S20**). These findings suggest that there are multiple pathways leading to individual differences in episodic memory in CU older adults, including elevated plasma $pTau_{181}$ with age that is linked to reduced memory through reduced neural selectivity, and reduced top-down attention that reduces episodic memory via decreased neural selectivity.

## Supplementary Methods

### MRI data preprocessing

Results included in this manuscript come from preprocessing performed using fMRIPrep 23.0.0rc0 (*101*) (RRID:SCR_016216), which is based on Nipype 1.8.5 (*102*, *103*) (RRID:SCR_002502). The below is an edited version of text automatically generated by fMRIPrep.

***Preprocessing of B0 inhomogeneity mappings.*** A total of two fieldmaps were found available within the input BIDS structure. A B0-nonuniformity map (or fieldmap) was estimated based on two (or more) echo-planar imaging (EPI) references with topup (*118*) (FSL 6.0.5.1:57b01774).

***Anatomical data preprocessing.*** One T1-weighted (T1w) images was collected in the current study. The T1w image was corrected for intensity non-uniformity (INU) with N4BiasFieldCorrection (*119*), distributed with ANTs 2.3.3 (*120*) (RRID:SCR_004757), and used as T1w-reference throughout the workflow. The T1w-reference was then skull-stripped with a Nipype implementation of the antsBrainExtraction.sh workflow (from ANTs), using OASIS30ANTs as target template. Brain tissue segmentation of cerebrospinal fluid (CSF), white-matter (WM) and gray-matter (GM) was performed on the brain-extracted T1w using fast (FSL 6.0.5.1:57b01774, RRID:SCR_002823) (*121*). Brain surfaces were reconstructed using recon-all (FreeSurfer 7.3.2, RRID:SCR_001847) (*122*), and the brain mask estimated previously was refined with a custom variation of the method to reconcile ANTs-derived and FreeSurfer-derived segmentations of the cortical gray-matter of Mindboggle (RRID:SCR_002438) (*123*). Grayordinate “dscalar” files (*104*) containing 91k samples were also generated using the highest-resolution *fsaverage* as an intermediate standardized surface space. Volume-based spatial normalization to two standard spaces (MNI152NLin2009cAsym, MNI152NLin6Asym) was performed through nonlinear registration with *antsRegistration* (ANTs 2.3.3), using brain-extracted versions of both T1w reference and the T1w template. The following templates were selected for spatial normalization: ICBM 152 Nonlinear Asymmetrical template version 2009c (RRID:SCR_008796 (*124*); TemplateFlow ID: MNI152NLin2009cAsym), FSL’s MNI ICBM 152 non-linear 6th Generation Asymmetric Average Brain Stereotaxic Registration Model (RRID:SCR_002823 (*125*); TemplateFlow ID: MNI152NLin6Asym).

***Functional data preprocessing.*** For each of the 10 BOLD runs per participant (5 encoding and 5 retrieval), the following preprocessing was performed. First, a reference volume and its skull-stripped version were generated using a custom methodology of fMRIPrep. Head-motion parameters with respect to the BOLD reference (transformation matrices, and six corresponding rotation and translation parameters) were estimated before spatiotemporal filtering using mcflirt (*126*) (FSL 6.0.5.1:57b01774). The estimated fieldmap was then aligned with rigid-registration to the target EPI (echo-planar imaging) reference run. The field coefficients were mapped on to the reference EPI using the transform. BOLD runs were slice-time corrected to 0.952s (0.5 of slice acquisition range 0s-1.9s) using 3dTshift from AFNI (*127*) (RRID:SCR_005927). The BOLD reference was then co-registered to the T1w reference using bbregister (FreeSurfer) which implements boundary-based registration (*128*). Co-registration was configured with six degrees of freedom.

Several confounds were calculated based on the preprocessed BOLD: framewise displacement (FD), DVARS and three region-wise global signals. FD was computed using two formulations, following Power (*129*) (absolute sum of relative motions) and Jenkinson (*126*) (relative root mean square displacement between affines). FD and DVARS were calculated for each functional run, using their implementations in Nipype (following the definitions by (*129*)). The three global signals were extracted within the CSF, WM, and whole-brain masks. Additionally, a set of physiological regressors were extracted to allow for component-based noise correction (*130*) (CompCor). Principal components were estimated after high-pass filtering the preprocessed BOLD time-series (using a discrete cosine filter with 128s cut-off) for the two CompCor variants: temporal (tCompCor) and anatomical (aCompCor). tCompCor components were then calculated from the top 2% variable voxels within the brain mask. For aCompCor, three probabilistic masks (CSF, WM and combined CSF+WM) were generated in anatomical space. The implementation differed from that of Behzadi et al. (*130*) in that instead of eroding the masks by two pixels on BOLD space, a mask of pixels that likely contain a volume fraction of GM was subtracted from the aCompCor masks. This mask was obtained by dilating a GM mask extracted from the FreeSurfer's aseg segmentation, and it ensured components were not extracted from voxels containing a minimal fraction of GM. Finally, these masks were resampled into BOLD space and binarized by thresholding at 0.99 (as in the original implementation). Components were also

calculated separately within the WM and CSF masks. For each CompCor decomposition, the k components with the largest singular values were retained, such that the retained components' time-series were sufficient to explain 50 percent of variance across the nuisance mask (CSF, WM, combined, or temporal). The remaining components were dropped from consideration.

The head-motion estimates calculated in the correction step were also placed within the corresponding confounds file. The confound time-series derived from head motion estimates and global signals were expanded with the inclusion of temporal derivatives and quadratic terms for each (*131*). Frames that exceeded a threshold of 0.9 mm FD or 3.0 standardized DVARS were annotated as motion outliers. Additional nuisance time-series were calculated by means of principal components analysis of the signal found within a thin band (crown) of voxels around the edge of the brain, as proposed by (*132*). The BOLD time-series were resampled into standard space, generating a preprocessed BOLD run in MNI152NLin2009cAsym space. First, a reference volume and its skull-stripped version were generated using a custom methodology of fMRIPrep. The BOLD time-series were resampled onto the following surfaces (FreeSurfer reconstruction nomenclature): fsnative, fsaverage5, fsaverage. Grayordinates files (*104*) containing 91k samples were also generated using the highest-resolution fsaverage as intermediate standardized surface space. All resamplings can be performed with a single interpolation step by composing all the pertinent transformations (i.e. head-motion transform matrices, susceptibility distortion correction when available, and co-registrations to anatomical and output spaces). Gridded (volumetric) resamplings were performed using antsApplyTransforms (ANTs), configured with Lanczos interpolation to minimize the smoothing effects of other kernels (*133*). Non-gridded (surface) resamplings were performed using mri_vol2surf (FreeSurfer).

Many internal operations of fMRIPrep use Nilearn 0.9.1 (RRID:SCR_001362) (*134*), mostly within the functional processing workflow. For more details of the pipeline, see the section corresponding to workflows in fMRIPrep's documentation.

**ROI-based neural selectivity by subsequent post-scan recall test**

To investigate whether higher neural selectivity at encoding enhances subsequent exemplar-specific recall, we conducted an independent general linear model in which in-scanner associative

hit events were categorized based on post-scan cued recall responses. Specifically, trials were classified as exemplar-specific hits, category-only hits, or misses. Non-associative hit trials were categorized similarly.

After model fitting, we extracted neural selectivity for each ROI, focusing on exemplar-specific and category-only hits within in-scanner associative hit trials. On average, 40% (SD = 18%; mean n = 16.69 ± 9.17 trials) of face-related and 49% (SD = 19%; mean n = 17.35 ± 9.89 trials) of place-related associative hit trials were classified as exemplar-specific hits. To ensure reliable estimates, participants with fewer than three trials in a given condition were excluded, resulting in a final sample of 107 participants.

A linear mixed-effects model (LMM) was then used to assess the effect of accuracy (Exemplar-Specific Hit vs. Category-Only Hit) on neural selectivity, including an Accuracy × ROI interaction term. Subject was modeled as a random intercept to account for individual differences.

## Supplementary tables

**Table S1.** Cortical brain regions exhibit significant category effects (n = 166; related to **Fig. 1B**).

| Contrast | Label index | Area ($mm^2$) | DKT_overlap |
|---|---|---|---|
| **Face > Place** | 1 | 1421.36 | L_precuneus (74.1%); L_isthmuscingulate (25.9%); |
| | 2 | 4393.79 | L_superiorfrontal (63.2%); L_medialorbitofrontal (13.1%); L_rostralanteriorcingulate (10.6%); L_rostralmiddlefrontal (6.0%); L_frontalpole (5.5%); |
| | 3 | 11917.38 | L_superiortemporal (23.7%); L_middletemporal (15.9%); L_inferiorparietal (13.3%); L_bankssts (7.3%); L_fusiform (6.4%); L_lateraloccipital (6.2%); L_lateralorbitofrontal (5.5%); |
| | 4 | 245.14 | L_lingual (100.0%); |
| | 5 | 1315.14 | R_precuneus (76.1%); R_isthmuscingulate (23.9%); |
| | 6 | 20183.86 | R_superiorfrontal (16.7%); R_middletemporal (13.2%); R_inferiorparietal (10.6%); R_superiortemporal (10.1%); R_fusiform (6.3%); R_medialorbitofrontal (6.0%); R_lateraloccipital (6.0%); |
| | 7 | 408.96 | R_lingual (100.0%); |
| **Face < Place** | 1 | 41115.71 | L_superiorparietal (10.9%); L_precentral (8.8%); L_lateraloccipital (8.0%); L_superiorfrontal (7.8%); L_rostralmiddlefrontal (6.8%); L_inferiorparietal (6.8%); L_precuneus (6.2%); L_inferiortemporal (5.4%); L_supramarginal (5.1%); |
| | 2 | 2969.92 | R_precentral (39.4%); R_parsopercularis (31.1%); R_insula (18.1%); R_lateralorbitofrontal (5.2%); |
| | 3 | 22558.92 | R_superiorparietal (19.8%); R_lateraloccipital (13.8%); R_inferiorparietal (11.5%); R_precuneus (10.8%); R_lingual (10.1%); R_supramarginal (9.6%); R_fusiform (7.2%); |
| | 4 | 4296.19 | R_superiorfrontal (62.8%); R_caudalmiddlefrontal (17.2%); R_precentral (11.9%); R_caudalanteriorcingulate (6.4%); |
| | 5 | 1466.17 | R_inferiortemporal (82.4%); R_middletemporal (15.7%); |
| | 6 | 2170.04 | R_rostralmiddlefrontal (90.5%); |
| | 7 | 169.13 | R_superiortemporal (100.0%); |

Note: DKT = Desikan-Killiany-Tourville; This table was generated using ciftify_statclust_report (https://github.com/edickie/ciftify/blob/master/ciftify/bin/ciftify_statclust_report.py).

**Table S2.** Cortical brain regions exhibit significant subsequent memory effects (n = 166; related to **Fig. 1C**).

| Contrast | Label index | Area ($mm^2$) | DKT_overlap |
|---|---|---|---|
| **Rem > Forg** | 1 | 85334.44 | R_rostralmiddlefrontal (6.4%); R_superiorfrontal (5.8%); L_rostralmiddlefrontal (5.6%); |
| | 2 | 55933.59 | L_superiorfrontal (11.8%); L_lateraloccipital (8.6%); L_precentral (7.9%); L_superiorparietal (7.2%); L_fusiform (6.1%); L_inferiorparietal (5.5%); L_postcentral (5.3%); |
| | 3 | 42206.97 | R_lateraloccipital (11.4%); R_superiorparietal (8.6%); R_precentral (8.1%); R_fusiform (7.9%); R_superiorfrontal (6.3%); R_lingual (6.1%); R_inferiorparietal (5.8%); |
| **Rem < Forg** | 1 | 2411.96 | L_superiortemporal (77.0%); L_transversetemporal (13.9%); L_insula (8.9%); |
| | 2 | 61.94 | L_lateralorbitofrontal (48.4%); L_rostralmiddlefrontal (44.5%); L_parsorbitalis (7.0%); |
| | 3 | 2764.19 | R_inferiorparietal (70.2%); R_supramarginal (29.8%); |
| | 4 | 1269.58 | R_rostralmiddlefrontal (80.4%); R_parstriangularis (10.5%); R_parsorbitalis (8.3%); |
| | 5 | 601.97 | R_medialorbitofrontal (53.1%); R_rostralanteriorcingulate (44.5%); |
| | 6 | 1103.66 | R_precuneus (88.6%); R_superiorparietal (7.9%); |
| | 7 | 1824.29 | R_superiortemporal (86.7%); R_transversetemporal (7.4%); R_insula (5.9%); |

Note: DKT = Desikan-Killiany-Tourville; This table was generated using ciftify_statclust_report (https://github.com/edickie/ciftify/blob/master/ciftify/bin/ciftify_statclust_report.py).

**Table S3.** Summary table of the LMM of age × memory (Rem vs Forg) × region (Face- vs Place-selective regions) predicting neural selectivity (n = 156; related to **Fig. 3A**).

| Neural selectivity ~ (age+educ+sex)*memory*region + (1\|subject) | | | | |
|---|---|---|---|---|
| **term** | **NumDF** | **DenDF** | **F** | **p** |
| **age** | **1** | **152** | **7.355** | **0.007** |
| educ | 1 | 152 | 1.819 | 0.179 |
| sex | 1 | 152 | 7.938 | 0.005 |
| **memory** | **1** | **456** | **14.76** | **0** |
| **region** | **1** | **456** | **8.588** | **0.004** |
| **age:memory** | **1** | **456** | **10.25** | **0.001** |
| educ:memory | 1 | 456 | 0.56 | 0.455 |
| sex:memory | 1 | 456 | 8.608 | 0.004 |
| **age:region** | **1** | **456** | **6.756** | **0.01** |
| educ:region | 1 | 456 | 0.086 | 0.77 |
| sex:region | 1 | 456 | 0.15 | 0.699 |
| memory:region | 1 | 456 | 0.002 | 0.966 |
| age:memory:region | 1 | 456 | 0.323 | 0.57 |
| educ:memory:region | 1 | 456 | 1.219 | 0.27 |
| sex:memory:region | 1 | 456 | 0.148 | 0.701 |

Note: NumDF = numerator degree of freedom, DenDF = denominator degree of freedom, educ = education.

**Table S4.** Summary table of the LMM of age × category (Face vs Place) × region (Face- vs Place-selective regions) predicting preferred/non-preferred neural activity on remembered trials (n = 156; related to **Fig. 3B**).

| Neural activity ~ (age+educ+sex)*category*region + (1\|subject) | | | | |
|---|---|---|---|---|
| **term** | **NumDF** | **DenDF** | **F** | **p** |
| **age** | **1** | **152** | **6.306** | **0.013** |
| sex | 1 | 152 | 11.146 | 0.001 |
| educ | 1 | 152 | 1.124 | 0.291 |
| category | 1 | 456 | 1.608 | 0.205 |
| region | 1 | 456 | 1.684 | 0.195 |
| age:category | 1 | 456 | 1.042 | 0.308 |
| sex:category | 1 | 456 | 0.075 | 0.785 |
| educ:category | 1 | 456 | 0.533 | 0.466 |
| age:region | 1 | 456 | 0.245 | 0.621 |
| sex:region | 1 | 456 | 23.177 | 0 |
| educ:region | 1 | 456 | 0.775 | 0.379 |
| **category:region** | **1** | **456** | **8.449** | **0.004** |
| **age:category:region** | **1** | **456** | **4.838** | **0.028** |
| sex:category:region | 1 | 456 | 4.653 | 0.032 |
| educ:category:region | 1 | 456 | 0.402 | 0.527 |

Note: NumDF = numerator degree of freedom, DenDF = denominator degree of freedom, educ = education.

**Table S5.** Summary table of the LMM of DAN SME × memory (Rem vs Forg) × region (Face- vs Place-selective regions) predicting neural selectivity (n = 156; related to **Fig. 3C**).

| Neural selectivity ~ sme_dan * memory * region + age + sex + educ + (1\|subject) | | | | |
|---|---|---|---|---|
| **term** | **NumDF** | **DenDF** | **F** | **p** |
| **sme_dan** | **1** | **151** | **9.632** | **0.002** |
| memory | 1 | 462 | 46.826 | 0 |
| region | 1 | 462 | 41.504 | 0 |
| age | 1 | 151 | 5.911 | 0.016 |
| sex | 1 | 151 | 6.645 | 0.011 |
| educ | 1 | 151 | 1.174 | 0.28 |
| **sme_dan:memory** | **1** | **462** | **18.92** | **0** |
| sme_dan:region | 1 | 462 | 1.732 | 0.189 |
| **memory:region** | **1** | **462** | **4.165** | **0.042** |
| sme_dan:memory:region | 1 | 462 | 1.318 | 0.252 |

Note: NumDF = numerator degree of freedom, DenDF = denominator degree of freedom, educ = education, sme_dan = subsequent memory effect in dorsal attention network.

**Table S6.** Summary table of the LMM of DAN SME × category (Face vs Place) × region (Face- vs Place-selective regions) predicting preferred/non-preferred neural activity on remembered trials (n = 156; related to **Fig. 3D**).

| Neural activity ~ sme_dan * category * region + age + sex + educ + (1\|subject) | | | | |
|---|---|---|---|---|
| **term** | **NumDF** | **DenDF** | **F** | **p** |
| sme_dan | 1 | 151 | 72.652 | 0 |
| category | 1 | 462 | 0.162 | 0.688 |
| region | 1 | 462 | 211.289 | 0 |
| age | 1 | 151 | 4.386 | 0.038 |
| sex | 1 | 151 | 10.2 | 0.002 |
| educ | 1 | 151 | 0.216 | 0.643 |
| sme_dan:category | 1 | 462 | 0.722 | 0.396 |
| sme_dan:region | 1 | 462 | 14.059 | 0 |
| category:region | 1 | 462 | 245.168 | 0 |
| **sme_dan:category:region** | **1** | **462** | **6.618** | **0.01** |
| **Region = Face-selective ROIs** | | | | |
| sme_dan | 1 | 222.05 | 86.651 | 0 |
| category | 1 | 462 | 230.058 | 0 |
| age | 1 | 151 | 4.386 | 0.038 |
| sex | 1 | 151 | 10.2 | 0.002 |
| educ | 1 | 151 | 0.216 | 0.643 |
| sme_dan:category | 1 | 462 | 1.484 | 0.224 |
| **Region = Place-selective ROIs** | | | | |
| sme_dan | 1 | 222.05 | 37.332 | 0 |
| category | 1 | 462 | 236.072 | 0 |
| age | 1 | 151 | 4.386 | 0.038 |
| sex | 1 | 151 | 10.2 | 0.002 |
| educ | 1 | 151 | 0.216 | 0.643 |
| **sme_dan:category** | **1** | **462** | **5.856** | **0.016** |

Note: NumDF = numerator degree of freedom, DenDF = denominator degree of freedom, educ = education, sme_dan = subsequent memory effect in dorsal attention network.

**Table S7.** Summary table of the LMM of plasma assay (log-transformed $pTau_{181}$ and $A\beta_{42}/A\beta_{40}$) × memory (Rem vs Forg) × region (Face- vs Place-selective regions) predicting neural selectivity (related to **Fig. 3E**).

| Plasma biomarkers | term | NumDF | DenDF | F | p |
|---|---|---|---|---|---|
| *Neural selectivity ~ pTau181*memory*region + age + sex + educ + (1\|subject)* | | | | | |
| pTau181 (log) n = 137 | pTau181 | 1 | 132 | 0.266 | 0.607 |
| | memory | 1 | 405 | 69.426 | 0 |
| | region | 1 | 405 | 72.832 | 0 |
| | age | 1 | 132 | 5.937 | 0.016 |
| | sex | 1 | 132 | 5.406 | 0.022 |
| | educ | 1 | 132 | 0.809 | 0.37 |
| | pTau181:memory | 1 | 405 | 3.561 | 0.06 |
| | **pTau181:region** | **1** | **405** | **14.797** | **0** |
| | memory:region | 1 | 405 | 0.511 | 0.475 |
| | pTau181:memory:region | 1 | 405 | 0.037 | 0.847 |
| *Neural selectivity ~ AB42/AB40*memory*region + age + sex + educ + (1\|subject)* | | | | | |
| AB42/AB40 n = 136 | AB42/AB40 | 1 | 131 | 3.038 | 0.084 |
| | memory | 1 | 402 | 0.002 | 0.968 |
| | region | 1 | 402 | 0.06 | 0.807 |
| | age | 1 | 131 | 6.531 | 0.012 |
| | sex | 1 | 131 | 4.639 | 0.033 |
| | educ | 1 | 131 | 0.379 | 0.539 |
| | AB42/AB4:memory | 1 | 402 | 2.377 | 0.124 |
| | AB42/AB40:region | 1 | 402 | 2.311 | 0.129 |
| | memory:region | 1 | 402 | 0.317 | 0.574 |
| | AB42/AB40:memory:region | 1 | 402 | 0.545 | 0.461 |

Note: NumDF = numerator degree of freedom, DenDF = denominator degree of freedom, educ = education.

**Table S8.** Summary table of the LMM of plasma $pTau_{181}$ × category (Face vs Place) predicting preferred/non-preferred neural activity on remembered trials in place-selective regions (n = 137; related to **Fig. 3F**).

Neural activity ~ pTau181 * category + age + sex + educ + (1|subj_id)

| term | NumDF | DenDF | F | p |
|---|---|---|---|---|
| pTau181 | 1 | 132 | 0.175 | 0.676 |
| category | 1 | 135 | 260.506 | 0 |
| age | 1 | 132 | 8.416 | 0.004 |
| sex | 1 | 132 | 2.132 | 0.147 |
| educ | 1 | 132 | 0.194 | 0.66 |
| **pTau181:category** | **1** | **135** | **10.392** | **0.002** |

Note: NumDF = numerator degree of freedom, DenDF = denominator degree of freedom, educ = education.

**Table S9.** Summary table of the LMM of CSF assay ($pTau_{181}$ and $A\beta_{42}/A\beta_{40}$) × memory (Rem vs Forg) × region (Face- vs Place-selective regions) predicting neural selectivity (n = 115; related to **Fig. S5**).

| CSF biomarkers | term | NumDF | DenDF | F | p |
|---|---|---|---|---|---|
| *Neural selectivity ~ pTau181*memory*region + age + sex + educ + (1\|subject)* | | | | | |
| pTau181 (log) | pTau181 | 1 | 110 | 0.263 | 0.609 |
| | memory | 1 | 339 | 4.599 | 0.033 |
| | region | 1 | 339 | 9.862 | 0.002 |
| | age | 1 | 110 | 5.075 | 0.026 |
| | sex | 1 | 110 | 5.775 | 0.018 |
| | educ | 1 | 110 | 1.274 | 0.261 |
| | pTau181:memory | 1 | 339 | 0.771 | 0.381 |
| | **pTau181:region** | **1** | **339** | **4.657** | **0.032** |
| | memory:region | 1 | 339 | 0.003 | 0.954 |
| | pTau181:memory:region | 1 | 339 | 0.066 | 0.797 |
| *Neural selectivity ~ AB42/AB40*memory*region + age + sex + educ + (1\|subject)* | | | | | |
| AB42/AB40 | AB42/AB40 | 1 | 110 | 0.052 | 0.821 |
| | memory | 1 | 339 | 5.003 | 0.026 |
| | region | 1 | 339 | 0.012 | 0.914 |
| | age | 1 | 110 | 4.881 | 0.029 |
| | sex | 1 | 110 | 6.077 | 0.015 |
| | educ | 1 | 110 | 1.283 | 0.26 |
| | AB42/AB4:memory | 1 | 339 | 0.208 | 0.649 |
| | **AB42/AB40:region** | **1** | **339** | **4.228** | **0.041** |
| | memory:region | 1 | 339 | 0.576 | 0.448 |
| | AB42/AB40:memory:region | 1 | 339 | 0.125 | 0.723 |

Note: NumDF = numerator degree of freedom, DenDF = denominator degree of freedom, educ = education.

**Table S10.** Summary of hierarchical linear regression models predicting neural selectivity (n = 137; related to **Figs. 4B & C**).

| | **Formula** | **Excluded regressors** | **$R^2$** |
|---|---|---|---|
| Full model | NS ~ DAN + Age + AD + Sex + Educ | -- | $0.259^{***}$ |
| 1a | NS ~ Age + AD + Sex + Educ | DAN | $0.165^{***}$ |
| 1b | NS ~ DAN + AD + Sex + Educ | Age | $0.225^{***}$ |
| 1c | NS ~ DAN + Age + Sex + Educ | AD | $0.239^{***}$ |
| 2a | NS ~ DAN + Sex + Educ | Age & AD | $0.168^{***}$ |
| 2b | NS ~ AD + Sex + Educ | Age & DAN | $0.114^{**}$ |
| 2c | NS ~ Age + Sex + Educ | DAN & AD | $0.144^{***}$ |

Note: NS = neural selectivity, DAN = DAN SME (subsequent memory effect in dorsal attention network), AD = plasma $pTau_{181}$, Educ = years of education; $^{**}$ $p < .01$, $^{***}$ $p < .001$.

**Table S11.** Summary of hierarchical linear regression models predicting memory performance (n = 137; related to **Figs. 6B & C**).

| | **Formula** | **Excluded regressors** | **$R^2$** |
|---|---|---|---|
| Full model | Memory ~ NS + DAN + Age + AD + Sex + Educ | -- | $0.284^{***}$ |
| 1a | Memory ~ DAN + Age + AD + Sex + Educ | NS | $0.240^{***}$ |
| 1b | Memory ~ NS + Age + AD + Sex + Educ | DAN | $0.260^{***}$ |
| 1c | Memory ~ NS + DAN + AD + Sex + Educ | Age | $0.256^{***}$ |
| 1d | Memory ~ NS + DAN + Age + Sex + Educ | AD | $0.271^{***}$ |
| 2a | Memory ~ DAN + NS + Sex + Educ | Age & AD | $0.222^{***}$ |
| 3a | Memory ~ AD | NS, DAN, Age, Sex & Educ | $0.085^{***}$ |

Note: Memory = place associative *d'*, NS = neural selectivity, DAN = DAN SME (subsequent memory effect in dorsal attention network), AD = plasma $pTau_{181}$, Educ = years of education; $^{***}$ $p < .001$.

**Table S12.** Results from SEM for overall associative *d'* and face associative *d'* (n = 137).

| | label | β.raw | β.std | p | 95% Confidence Interval lower | 95% Confidence Interval upper |
|---|---|---|---|---|---|---|
| Overall associative *d'* | $a_1$ | 0.0094 | 0.4188 | 0.0000 | 0.0056 | 0.0131 |
| | $a_2$ | -0.0219 | -0.2107 | 0.0012 | -0.0347 | -0.0078 |
| | $d_{21}$ | -0.7311 | -0.1579 | 0.0134 | -1.3222 | -0.1696 |
| | $d_{23}$ | 0.562 | 0.3125 | 0.0000 | 0.3372 | 0.8194 |
| | $a_3$ | -0.0084 | -0.1459 | 0.0495 | -0.0170 | 0.0000 |
| | $b_1$ | -0.8145 | -0.1457 | 0.1757 | -1.9661 | 0.3924 |
| | $b_2$ | 0.1765 | 0.1462 | 0.0794 | -0.0071 | 0.3881 |
| | $b_3$ | 0.2481 | 0.1143 | 0.1517 | -0.1385 | 0.5558 |
| | $a_1b_1$ | -0.0077 | -0.0610 | 0.1736 | -0.0194 | 0.0032 |
| | $a_1d_{21}$ | -0.0069 | -0.0661 | 0.0151 | -0.0136 | -0.0022 |
| | $d_{21}b_2$ | -0.129 | -0.0231 | 0.1623 | -0.4033 | -0.0040 |
| | $a_1d_{21}b_2$ | -0.0012 | -0.0097 | 0.1653 | -0.0039 | -0.0001 |
| | $a_2b_2$ | -0.0039 | -0.0308 | 0.1351 | -0.0108 | -0.0001 |
| | $a_3b_3$ | -0.0021 | -0.0167 | 0.2632 | -0.0074 | 0.0005 |
| | $a_3d_{23}$ | -0.0047 | -0.0456 | 0.0659 | -0.0106 | -0.0002 |
| | $d_{23}b_2$ | 0.0992 | 0.0457 | 0.1176 | 0.0012 | 0.2605 |
| | $a_3d_{23}b_2$ | -0.0008 | -0.0067 | 0.2631 | -0.0033 | 0.0000 |
| | $c'$ | -0.0275 | -0.2190 | 0.0310 | -0.0510 | -0.0010 |
| | $c$ | -0.0432 | -0.3438 | 0.0002 | -0.0656 | -0.0213 |
| Face associative *d'* | $a_1$ | 0.0094 | 0.4188 | 0.0000 | 0.0057 | 0.0133 |
| | $a_2$ | -0.0219 | -0.2107 | 0.0019 | -0.0353 | -0.0081 |
| | $d_{21}$ | -0.7311 | -0.1579 | 0.0128 | -1.3219 | -0.1568 |
| | $d_{23}$ | 0.562 | 0.3125 | 0.0000 | 0.3402 | 0.8235 |
| | $a_3$ | -0.0084 | -0.1459 | 0.0504 | -0.0169 | -0.0001 |
| | $b_1$ | -0.4737 | -0.1012 | 0.3485 | -1.4552 | 0.5110 |
| | $b_2$ | 0.1238 | 0.1224 | 0.1491 | -0.0426 | 0.3008 |
| | $b_3$ | 0.2682 | 0.1474 | 0.0797 | -0.0411 | 0.5687 |
| | $a_1b_1$ | -0.0045 | -0.0424 | 0.3477 | -0.0141 | 0.0049 |
| | $a_1d_{21}$ | -0.0069 | -0.0661 | 0.0146 | -0.0134 | -0.0020 |
| | $d_{21}b_2$ | -0.0905 | -0.0193 | 0.2229 | -0.2858 | 0.0112 |
| | $a_1d_{21}b_2$ | -0.0009 | -0.0081 | 0.2275 | -0.0029 | 0.0001 |
| | $a_2b_2$ | -0.0027 | -0.0258 | 0.2059 | -0.0079 | 0.0006 |
| | $a_3b_3$ | -0.0023 | -0.0215 | 0.2092 | -0.0079 | 0.0000 |
| | $a_3d_{23}$ | -0.0047 | -0.0456 | 0.0700 | -0.0111 | -0.0003 |
| | $d_{23}b_2$ | 0.0696 | 0.0382 | 0.1930 | -0.0177 | 0.1973 |

| | | | | | |
|---|---|---|---|---|---|
| $a_3d_{23}b_2$ | -0.0006 | -0.0056 | 0.3357 | -0.0026 | 0.0001 |
| $c'$ | -0.0184 | -0.1745 | 0.0919 | -0.0384 | 0.0043 |
| $c$ | -0.0292 | -0.2778 | 0.0013 | -0.0466 | -0.0111 |

Note: β.std = standardized beta. $c'$ = direct effect, $c = c' + a_1b_1 + a_2b_2 + a_3b_3 + a_1d_{21}b_2 + a_3d_{23}b_2$ = total effect.

**Table S13.** Mediation results of age → neural selectivity → memory performance (overall, place, and face associative *d'*) (n = 156).

| | label | β.raw | β.std | pvalue | 95% Confidence Interval lower | upper |
|---|---|---|---|---|---|---|
| Place associative *d'* | *b* | 0.366 | 0.308 | <.001 | 0.202 | 0.551 |
| | *c'* | -0.028 | -0.229 | 0.009 | -0.05 | -0.008 |
| | *a* | -0.029 | -0.276 | <.001 | -0.041 | -0.015 |
| | ***ab*** | **-0.011** | **-0.085** | **0.003** | **-0.019** | **-0.005** |
| | *c'+ab* | -0.039 | -0.314 | <.001 | -0.061 | -0.017 |
| Face associative *d'* | *b* | 0.233 | 0.22 | 0.005 | 0.08 | 0.409 |
| | *c'* | -0.02 | -0.178 | 0.033 | -0.038 | -0.001 |
| | *a* | -0.029 | -0.276 | <.001 | -0.041 | -0.015 |
| | ***ab*** | **-0.007** | **-0.061** | **0.024** | **-0.014** | **-0.002** |
| | *c'+ab* | -0.026 | -0.239 | 0.003 | -0.043 | -0.009 |
| Overall associative *d'* | *b* | 0.259 | 0.21 | 0.006 | 0.08 | 0.444 |
| | *c'* | -0.033 | -0.253 | 0.003 | -0.053 | -0.011 |
| | *a* | -0.029 | -0.276 | <.001 | -0.042 | -0.016 |
| | ***ab*** | **-0.007** | **-0.058** | **0.023** | **-0.015** | **-0.002** |
| | *c'+ab* | -0.04 | -0.311 | <.001 | -0.061 | -0.018 |

Note: β.std = standardized beta. *c'* = direct effect, *ab* = indirect effect, *c'+ab* = total effect.

**Table S14.** Mediation results of DAN SME → neural selectivity → memory performance (overall, place, and face associative *d'*) (n = 156).

| | label | β.raw | β.std | p | 95% Confidence Interval lower | upper |
|---|---|---|---|---|---|---|
| Place associative *d'* | *b* | 0.299 | 0.251 | 0.001 | 0.136 | 0.490 |
| | *c'* | 0.345 | 0.159 | 0.028 | 0.015 | 0.641 |
| | *a* | 0.585 | 0.321 | <.001 | 0.373 | 0.840 |
| | ***ab*** | **0.175** | **0.081** | **0.005** | **0.080** | **0.336** |
| | *c'*+*ab* | 0.520 | 0.239 | 0.001 | 0.196 | 0.820 |
| Face associative *d'* | *b* | 0.188 | 0.178 | 0.028 | 0.024 | 0.364 |
| | *c'* | 0.229 | 0.119 | 0.122 | -0.065 | 0.518 |
| | *a* | 0.585 | 0.321 | <.001 | 0.375 | 0.842 |
| | ***ab*** | **0.110** | **0.057** | **0.061** | **0.018** | **0.249** |
| | *c'*+*ab* | 0.339 | 0.176 | 0.013 | 0.066 | 0.605 |
| Overall associative *d'* | *b* | 0.218 | 0.177 | 0.026 | 0.048 | 0.431 |
| | *c'* | 0.207 | 0.092 | 0.215 | -0.152 | 0.520 |
| | *a* | 0.585 | 0.321 | <.001 | 0.375 | 0.842 |
| | ***ab*** | **0.128** | **0.057** | **0.055** | **0.029** | **0.295** |
| | *c'*+*ab* | 0.335 | 0.149 | 0.036 | -0.007 | 0.624 |

Note: β.std = standardized beta. *c'* = direct effect, *ab* = indirect effect, *c'*+*ab* = total effect.

**Table S15.** Results from SEM for delayed recall composite score (n = 137).

| | label | β.raw | β.std | p | 95% Confidence Interval | |
|---|---|---|---|---|---|---|
| | | | | | lower | upper |
| Delayed recall composite score (Z) | $a_1$ | 0.0094 | 0.4188 | 0.0000 | 0.0057 | 0.0132 |
| | $a_2$ | -0.0219 | -0.2107 | 0.0019 | -0.0358 | -0.0078 |
| | $d_{21}$ | -0.7311 | -0.1579 | 0.0148 | -1.3118 | -0.1199 |
| | $d_{23}$ | 0.5620 | 0.3125 | 0.0000 | 0.3429 | 0.8252 |
| | $a_3$ | -0.0084 | -0.1459 | 0.0495 | -0.0170 | 0.0001 |
| | $b_1$ | -0.1847 | -0.0314 | 0.7553 | -1.4875 | 0.8491 |
| | $b_2$ | 0.2104 | 0.1653 | 0.0330 | 0.0113 | 0.3962 |
| | $b_3$ | 0.3503 | 0.1530 | 0.0477 | -0.0075 | 0.6874 |
| | $a_1b_1$ | -0.0017 | -0.0131 | 0.7594 | -0.0150 | 0.0085 |
| | $a_1d_{21}$ | -0.0069 | -0.0661 | 0.0168 | -0.0135 | -0.0019 |
| | $d_{21}b_2$ | -0.1538 | -0.0261 | 0.1273 | -0.4280 | -0.0089 |
| | $a_1d_{21}b_2$ | -0.0015 | -0.0109 | 0.1246 | -0.0042 | -0.0001 |
| | $a_2b_2$ | -0.0046 | -0.0348 | 0.0803 | -0.0113 | -0.0006 |
| | $a_3b_3$ | -0.0030 | -0.0223 | 0.1842 | -0.0094 | 0.0000 |
| | $a_3d_{23}$ | -0.0047 | -0.0456 | 0.0634 | -0.0104 | -0.0002 |
| | $d_{23}b_2$ | 0.1183 | 0.0517 | 0.0624 | 0.0106 | 0.2657 |
| | $a_3d_{23}b_2$ | -0.0010 | -0.0075 | 0.1852 | -0.0033 | 0.0000 |
| | $c'$ | -0.0367 | -0.2772 | 0.0030 | -0.0590 | -0.0106 |
| | $c$ | -0.0485 | -0.3660 | 0.0000 | -0.0700 | -0.0250 |

Note: β.std = standardized beta. $c'$ = direct effect, $c = c' + a_1b_1 + a_2b_2 + a_3b_3 + a_1d_{21}b_2 + a_3d_{23}b_2$ = total effect.

**Table S16.** Mediation results of age → neural selectivity → delayed recall composite score (n = 156).

| | | | | | 95% Confidence Interval | |
|---|---|---|---|---|---|---|
| | **label** | **β.raw** | **β.std** | **p** | **lower** | **upper** |
| Delayed recall composite score (Z) | *b* | 0.2764 | 0.2185 | 0.0023 | 0.0975 | 0.4553 |
| | *c′* | -0.0357 | -0.2708 | 0.0006 | -0.0558 | -0.0155 |
| | *a* | -0.0288 | -0.2761 | 0 | -0.0421 | -0.0156 |
| | ***ab*** | **-0.008** | **-0.0603** | **0.012** | **-0.0157** | **-0.0029** |
| | *c′+ab* | -0.0437 | -0.3311 | 0 | -0.0644 | -0.0218 |

Note: β.std = standardized beta. *c'* = direct effect, *ab* = indirect effect, *c′+ab* = total effect.

**Table S17.** Mediation results of top-down attention → neural selectivity → delayed recall composite score (n = 156).

| | | | | | 95% Confidence Interval | |
|---|---|---|---|---|---|---|
| | **label** | **β.raw** | **β.std** | **p** | **lower** | **upper** |
| Delayed recall composite score (Z) | *b* | 0.215 | 0.17 | 0.024 | 0.027 | 0.403 |
| | *c'* | 0.315 | 0.136 | 0.075 | -0.035 | 0.651 |
| | *a* | 0.585 | 0.321 | 0 | 0.371 | 0.831 |
| | ***ab*** | **0.126** | **0.055** | **0.049** | **0.022** | **0.28** |
| | *c'+ab* | 0.441 | 0.191 | 0.008 | 0.105 | 0.757 |

Note: β.std = standardized beta. *c'* = direct effect, *ab* = indirect effect, *c'+ab* = total effect.

**Table S18.** Results from SEM for mnemonic similarity task performance (n = 119).

| | label | β.raw | β.std | p | 95% Confidence Interval lower | upper |
|---|---|---|---|---|---|---|
| Lure/new d' similarity slope | $a_1$ | 0.0098 | 0.4388 | 0.0000 | 0.0059 | 0.0139 |
| | $a_2$ | -0.0194 | -0.1899 | 0.0070 | -0.0335 | -0.0053 |
| | $d_{21}$ | -0.6687 | -0.1454 | 0.0312 | -1.2623 | -0.0395 |
| | $d_{23}$ | 0.6146 | 0.3445 | 0.0000 | 0.3891 | 0.9081 |
| | $a_3$ | -0.0088 | -0.1540 | 0.0545 | -0.0181 | 0.0000 |
| | $b_1$ | -0.0640 | -0.1191 | 0.2502 | -0.1744 | 0.0405 |
| | $b_2$ | 0.0202 | 0.1730 | 0.0473 | -0.0001 | 0.0403 |
| | $b_3$ | 0.0220 | 0.1055 | 0.1620 | -0.0057 | 0.0569 |
| | $a_1b_1$ | -0.0006 | -0.0523 | 0.2736 | -0.0019 | 0.0003 |
| | $a_1d_{21}$ | -0.0065 | -0.0638 | 0.0295 | -0.0130 | -0.0011 |
| | $d_{21}b_2$ | -0.0135 | -0.0251 | 0.1719 | -0.0408 | 0.0000 |
| | $a_1d_{21}b_2$ | -0.0001 | -0.0110 | 0.1696 | -0.0004 | 0.0000 |
| | $a_2b_2$ | -0.0004 | -0.0328 | 0.1265 | -0.0010 | 0.0000 |
| | $a_3b_3$ | -0.0002 | -0.0162 | 0.2669 | -0.0008 | 0.0000 |
| | $a_3d_{23}$ | -0.0054 | -0.0531 | 0.0683 | -0.0123 | -0.0002 |
| | $d_{23}b_2$ | 0.0124 | 0.0596 | 0.0670 | 0.0004 | 0.0277 |
| | $a_3d_{23}b_2$ | -0.0001 | -0.0092 | 0.1809 | -0.0004 | 0.0000 |
| | $c'$ | -0.0030 | -0.2535 | 0.0102 | -0.0053 | -0.0007 |
| | $c$ | -0.0045 | -0.3750 | 0.0002 | -0.0068 | -0.0021 |
| Old/lure d' similarity slope | $a_1$ | 0.0098 | 0.4388 | 0.0000 | 0.0058 | 0.0138 |
| | $a_2$ | -0.0194 | -0.1899 | 0.0074 | -0.0329 | -0.0038 |
| | $d_{21}$ | -0.6687 | -0.1454 | 0.0294 | -1.2732 | -0.0434 |
| | $d_{23}$ | 0.6146 | 0.3445 | 0.0000 | 0.3841 | 0.8994 |
| | $a_3$ | -0.0088 | -0.1540 | 0.0551 | -0.0181 | 0.0002 |
| | $b_1$ | 0.0072 | 0.0136 | 0.8774 | -0.0861 | 0.0950 |
| | $b_2$ | 0.0204 | 0.1771 | 0.0655 | -0.0027 | 0.0407 |
| | $b_3$ | 0.0284 | 0.1380 | 0.1214 | -0.0039 | 0.0670 |
| | $a_1b_1$ | 0.0001 | 0.0060 | 0.8789 | -0.0009 | 0.0009 |
| | $a_1d_{21}$ | -0.0065 | -0.0638 | 0.0258 | -0.0129 | -0.0013 |
| | $d_{21}b_2$ | -0.0136 | -0.0257 | 0.2103 | -0.0443 | 0.0004 |
| | $a_1d_{21}b_2$ | -0.0001 | -0.0113 | 0.1960 | -0.0004 | 0.0000 |
| | $a_2b_2$ | -0.0004 | -0.0336 | 0.1402 | -0.0011 | 0.0000 |
| | $a_3b_3$ | -0.0003 | -0.0213 | 0.2253 | -0.0009 | 0.0000 |

| | | | | | |
|---|---|---|---|---|---|
| $a_3d_{23}$ | -0.0054 | -0.0531 | 0.0728 | -0.0125 | -0.0004 |
| $d_{23}b_2$ | 0.0125 | 0.0610 | 0.0987 | -0.0006 | 0.0297 |
| $a_3d_{23}b_2$ | -0.0001 | -0.0094 | 0.2192 | -0.0004 | 0.0000 |
| $c'$ | -0.0037 | -0.3120 | 0.0010 | -0.0058 | -0.0013 |
| $c$ | -0.0045 | -0.3816 | 0.0000 | -0.0064 | -0.0023 |

Note: β.std = standardized beta. $c'$ = direct effect, $c = c' + a_1b_1 + a_2b_2 + a_3b_3 + a_1d_{21}b_2 + a_3d_{23}b_2$ = total effect.

**Table S19.** Mediation results of age → neural selectivity → mnemonic similarity task performance (n = 135).

| | label | β.raw | β.std | p | 95% Confidence Interval | |
|---|---|---|---|---|---|---|
| | | | | | **lower** | **upper** |
| Lure/new *d′* similarity slope | *b* | 0.026 | 0.221 | 0.0039 | 0.0083 | 0.0439 |
| | *c′* | -0.0034 | -0.2845 | 0.0036 | -0.0057 | -0.0011 |
| | *a* | -0.0282 | -0.2746 | 0.0001 | -0.0416 | -0.0141 |
| | ***ab*** | **-0.0007** | **-0.0607** | **0.0187** | **-0.0015** | **-0.0002** |
| | *c′+ab* | -0.0042 | -0.3452 | 0.0003 | -0.0064 | -0.0018 |
| Old/lure *d′* similarity slope | *b* | 0.0285 | 0.246 | 0.0024 | 0.0093 | 0.0467 |
| | *c′* | -0.0032 | -0.2674 | 0.0014 | -0.0051 | -0.0012 |
| | *a* | -0.0282 | -0.2746 | 0 | -0.0415 | -0.0144 |
| | ***ab*** | **-0.0008** | **-0.0676** | **0.0165** | **-0.0016** | **-0.0002** |
| | *c′+ab* | -0.004 | -0.335 | 0 | -0.0058 | -0.002 |

Note: β.std = standardized beta. *c′* = direct effect, *ab* = indirect effect, *c′+ab* = total effect.

**Table S20.** Mediation results of top-down attention → neural selectivity → mnemonic similarity task performance (n = 135).

| | label | β.raw | β.std | p | 95% Confidence Interval | |
|---|---|---|---|---|---|---|
| | | | | | **lower** | **upper** |
| Lure/new *d′* similarity slope | *b* | 0.0221 | 0.1884 | 0.0236 | 0.0031 | 0.0418 |
| | *c′* | 0.0169 | 0.0804 | 0.2586 | -0.0085 | 0.0516 |
| | *a* | 0.6348 | 0.3548 | 0 | 0.4247 | 0.9107 |
| | ***ab*** | **0.014** | **0.0669** | **0.0388** | **0.0026** | **0.0294** |
| | *c′+ab* | 0.0309 | 0.1473 | 0.0415 | 0.0049 | 0.0647 |
| Old/lure *d′* similarity slope | *b* | 0.0228 | 0.1971 | 0.0253 | 0.0027 | 0.0431 |
| | *c′* | 0.025 | 0.1205 | 0.1563 | -0.0077 | 0.0627 |
| | *a* | 0.6348 | 0.3548 | 0 | 0.4141 | 0.9063 |
| | ***ab*** | **0.0145** | **0.0699** | **0.0483** | **0.002** | **0.0309** |
| | *c′+ab* | 0.0394 | 0.1905 | 0.0186 | 0.0087 | 0.0752 |

Note: β.std = standardized beta. *c'* = direct effect, *ab* = indirect effect, *c′+ab* = total effect.

**Supplementary Figures**

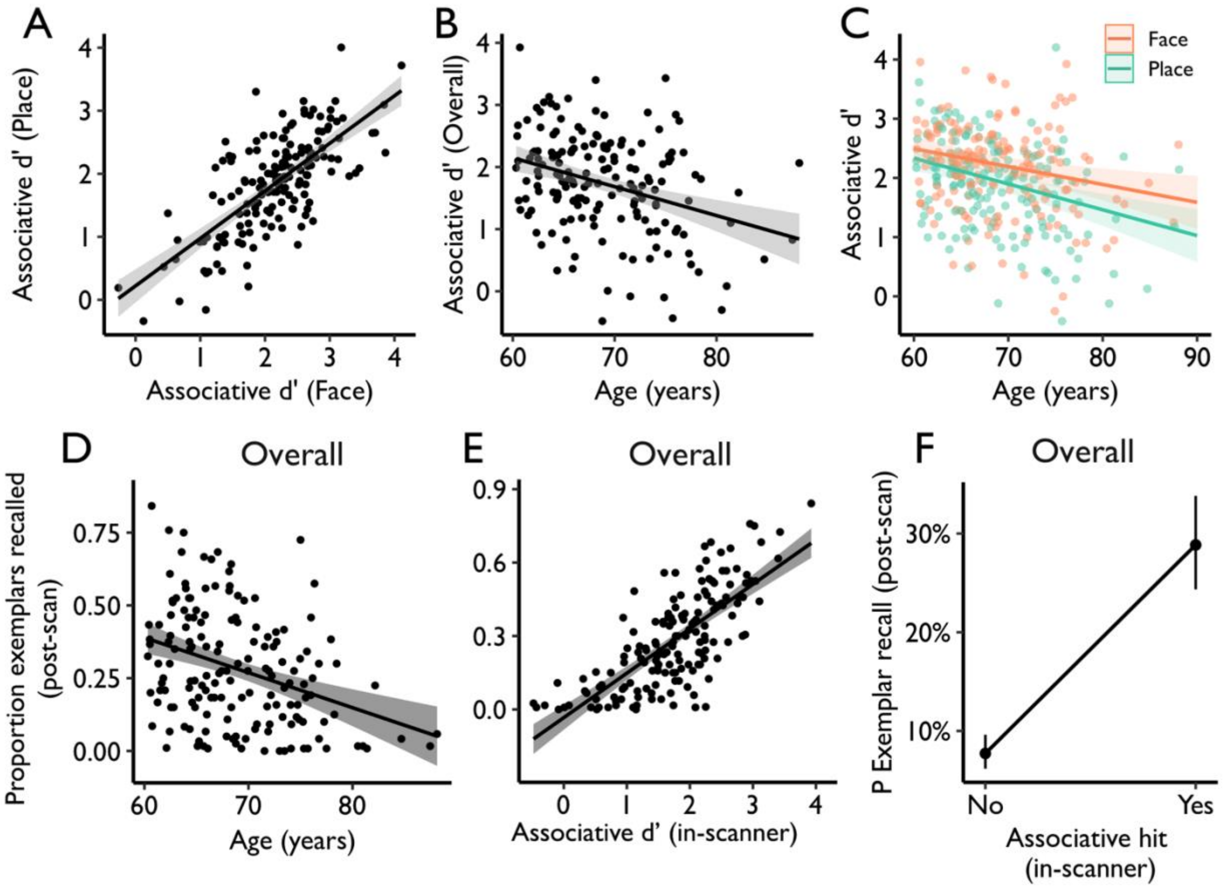


**Fig. S1. Behavioral results.** (A) Face and place associative memory were highly correlated ($R$ = 0.71, p < 0.001). Overall (skew = -.33, kurtosis = .16), face (skew = -.29, kurtosis = .58), and place (skew = -.26, kurtosis = .04) associative *d'* followed a normal-like distribution. (B) Overall ($\beta$ = -.044, $p$ < .001) and (C) face (warm color; $\beta$ = -.030, $p$ = .002) and place (cool color; $\beta$ = -.044, $p$ < .001) memory performance declined with age, controlling for sex and years of education. (D) Proportion correct recall of the specific exemplars during the post-scan cued recall also decreased with age (overall: $\beta$ = -.011, $p$ < .001) and highly correlated with in-scanner associative *d'* both (E) across-individuals (overall: $\beta$ = .170, $p$ < .001) and (F) within-individuals (overall: $\beta$ = 1.578, $p$ < .001).

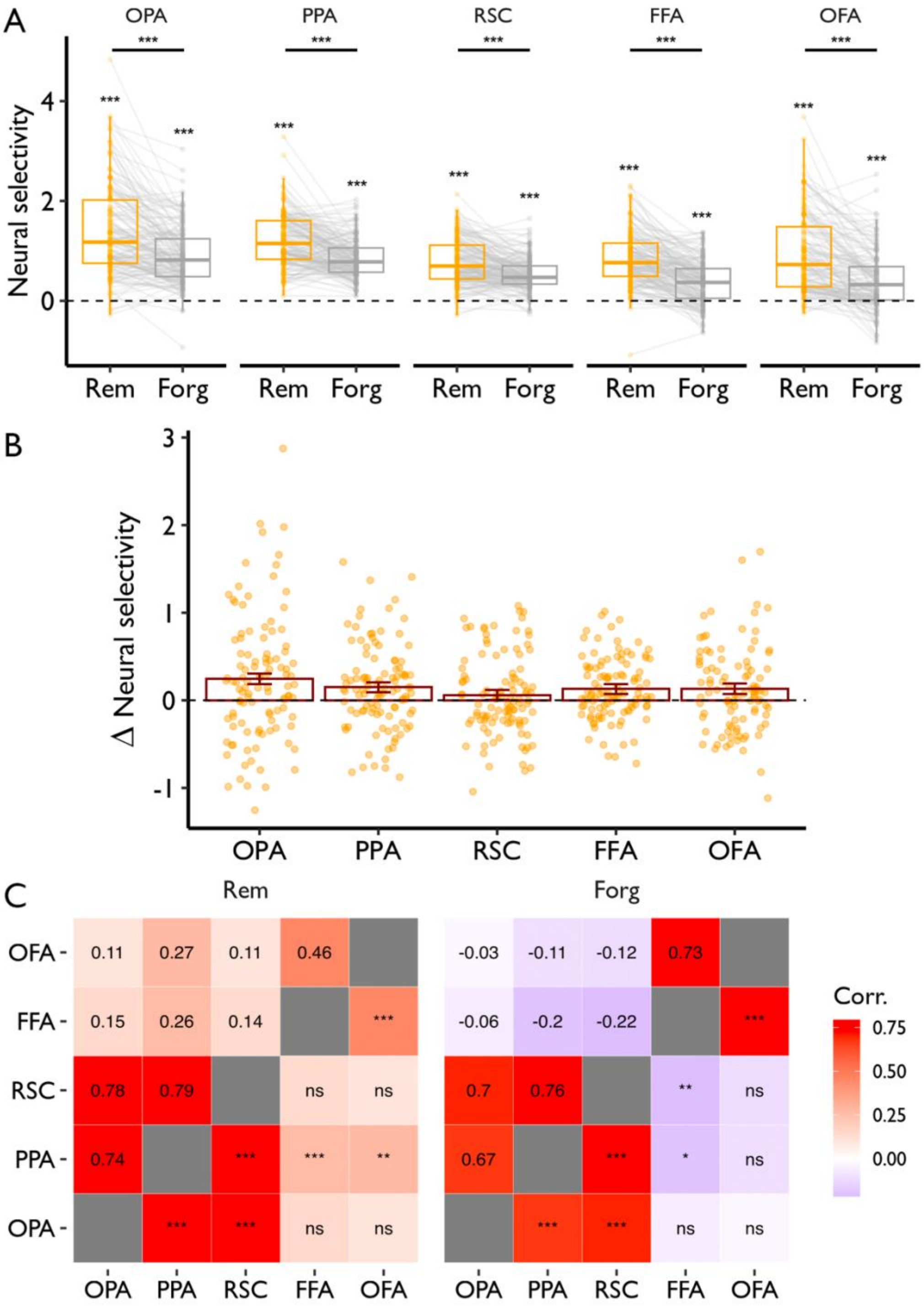


**Fig. S2. Neural selectivity varies with subsequent memory and remains more consistent within the same types of ROIs.** (A) Neural selectivity in study-specific ROIs was greater for subsequently remembered trials than for forgotten trials. (B) Among in-scanner associative hit trials (orange points in A), neural selectivity was significantly higher for trials for which

participants recalled the specific associated image (exemplar-specific hits) relative to trials for which they recalled the image category (category-only hits) during the post-scan test ($p < .001$). (C) Neural selectivity was correlated among place- (i.e., PPA, OPA, and RSC) or face-selective ROIs (i.e., FFA and OFA); the upper triangles show Pearson's correlations, and the lower triangles show significance; ns = non-significant, *$p < .05$, **$p < .01$, ***$p < .001$.

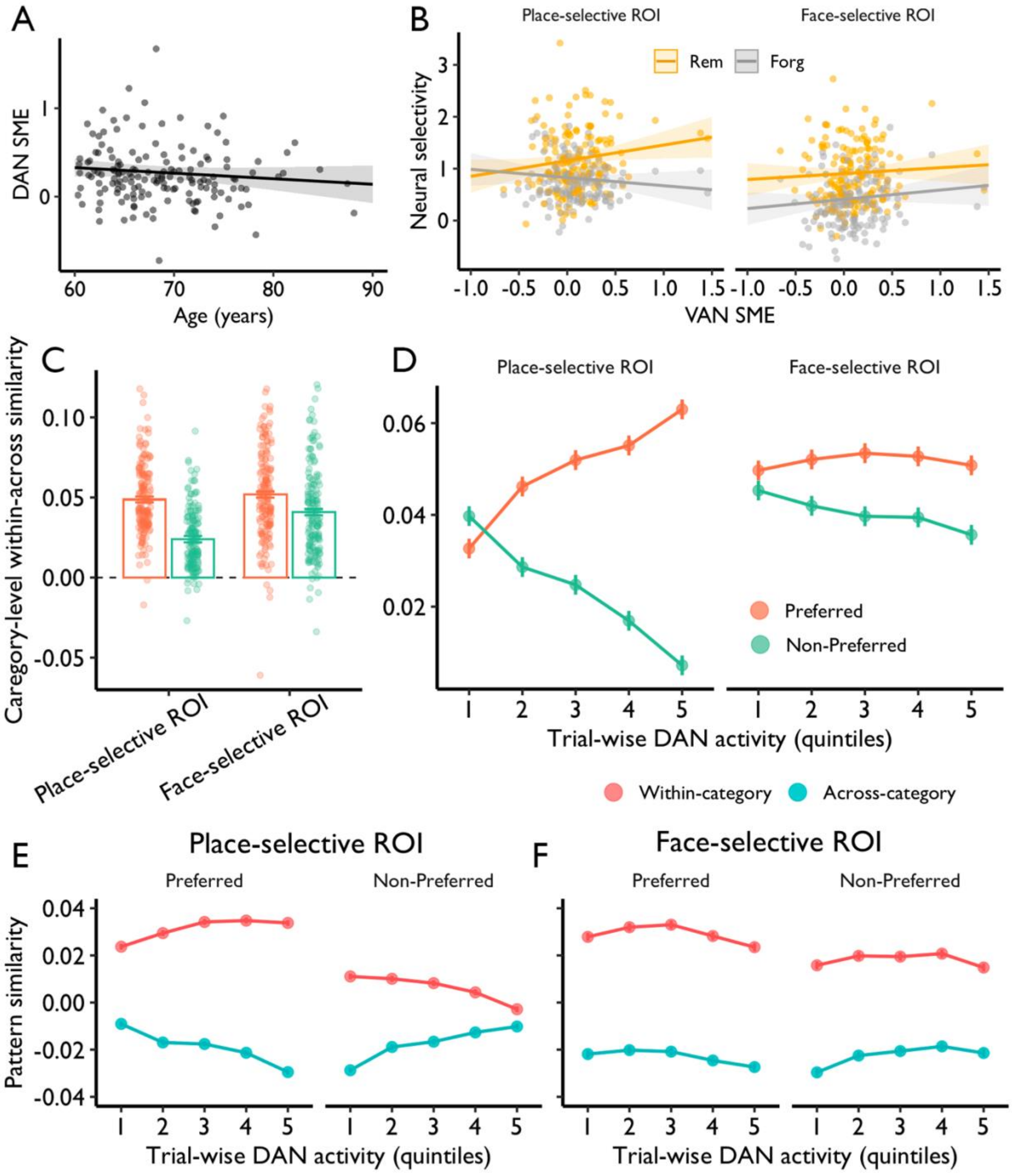


**Fig. S3. The effects of top-down and bottom-up attention on neural selectivity.** (A) Scatterplot of DAN SME by age. (B) Scatterplots of neural selectivity by VAN SME. (C) Trial-wise neural selectivity was greater for preferred categories than for non-preferred categories. (D) Trial-by-trial DAN fluctuations (restricted to subsequent associative hit trials, and thus controlling for memory)

predict trial-wise neural selectivity in place-selective regions. (E) DAN activity positively correlated with within-category similarity ($\beta$ = .005, $p_{Holm}$ < .001) and negatively correlated with across-category similarity ($\beta$ = -.008, $p_{Holm}$ < .001) for place trials (preferred category) in place-selective regions, while the opposite pattern was observed for face trials (non-preferred category; within-category: $\beta$ = -.006, $p_{Holm}$ < .001; across-category: $\beta$ = .008, $p_{Holm}$ < .001). (F) DAN activity was negatively related to both within- ($\beta$ = -.002, $p_{Holm}$ < .001) and across-category similarity ($\beta$ = -.002, $p_{Holm}$ = .003) for face trials (preferred category) but positively related to similarity for place trials (non-preferred category; within-category: $\beta$ = .0006, $p_{Holm}$ = .276; across-category: $\beta$ = .003, $p_{Holm}$ < .001) in face-selective regions. Error bars represent within-subject errors. DAN, dorsal attention network; VAN, ventral attention network; SME, subsequent memory effect.

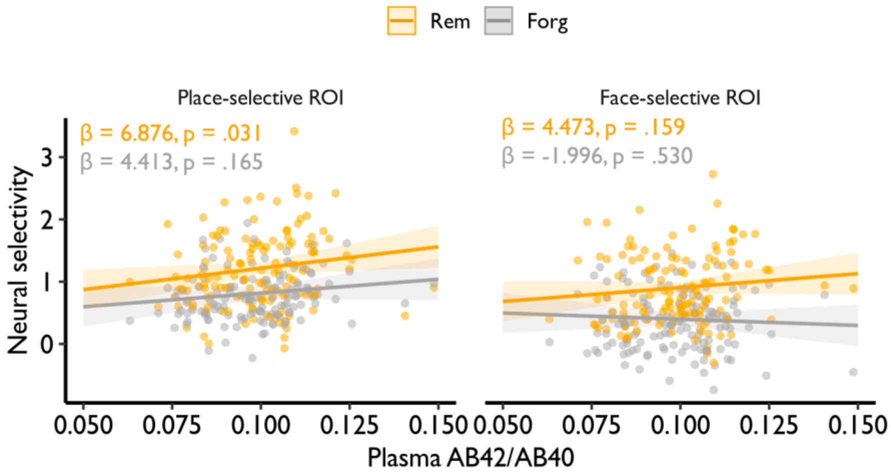


**Fig. S4. Relationship between neural selectivity and plasma $A\beta_{42}/A\beta_{40}$ (n = 136).**

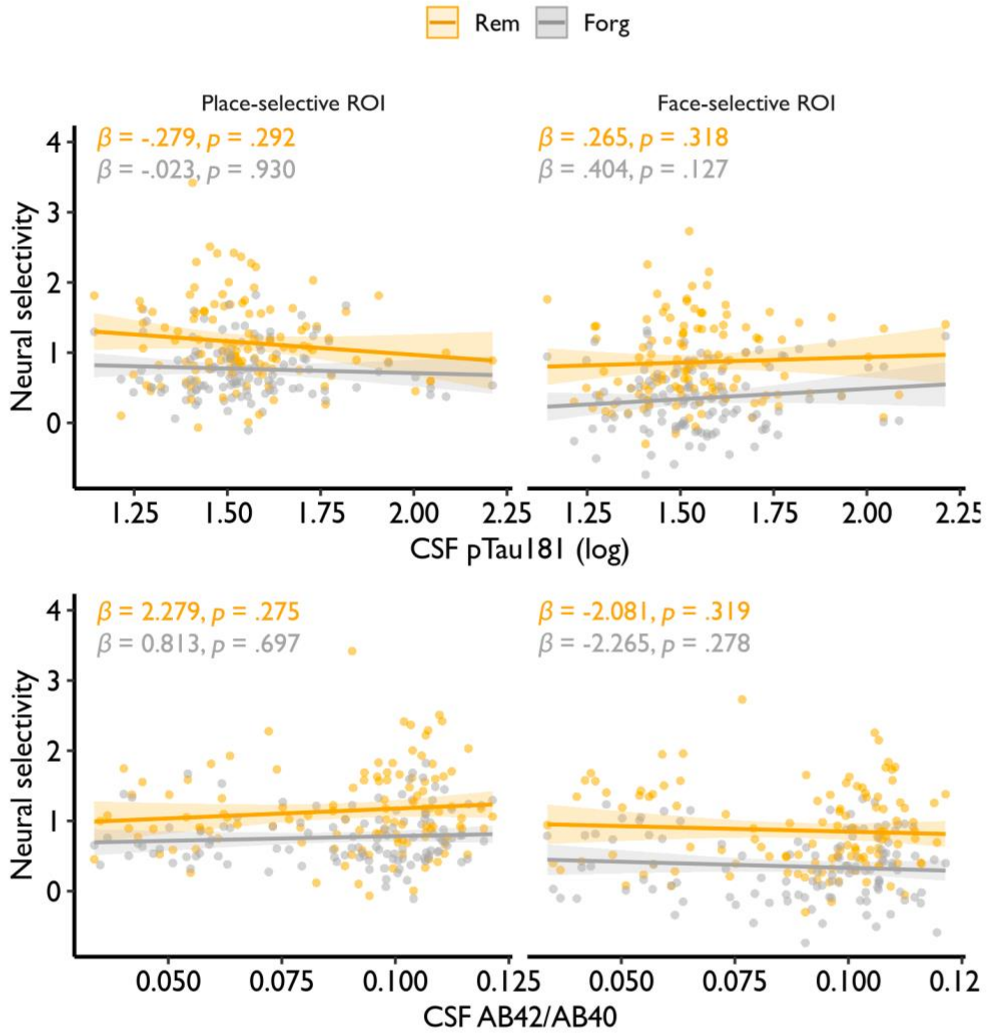


**Fig. S5. Relationship between neural selectivity and CSF AD biomarkers.** (top) $pTau_{181}$ and (bottom) $A\beta_{42}/A\beta_{40}$ (n = 115).

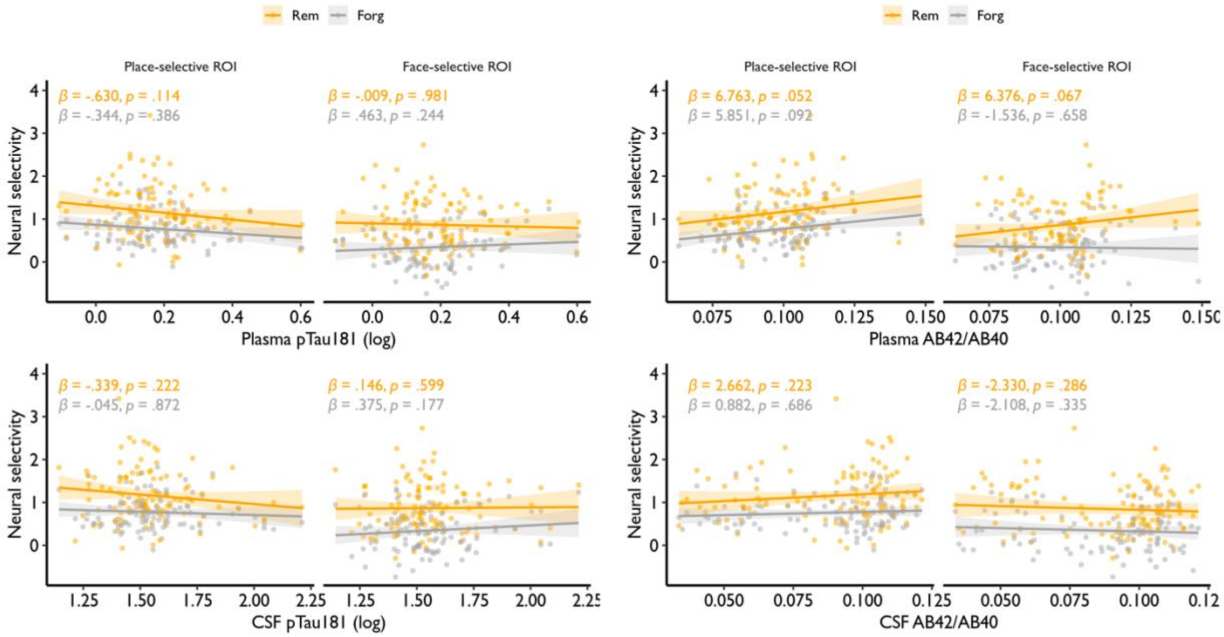


**Fig. S6. Relationship between neural selectivity and AD biomarkers in the subset of participants with both plasma and CSF.** Neural selectivity is plotted as a function of $pTau_{181}$ (n = 104) and $A\beta_{42}/A\beta_{40}$ (n = 103) in (top) plasma and (bottom) CSF.

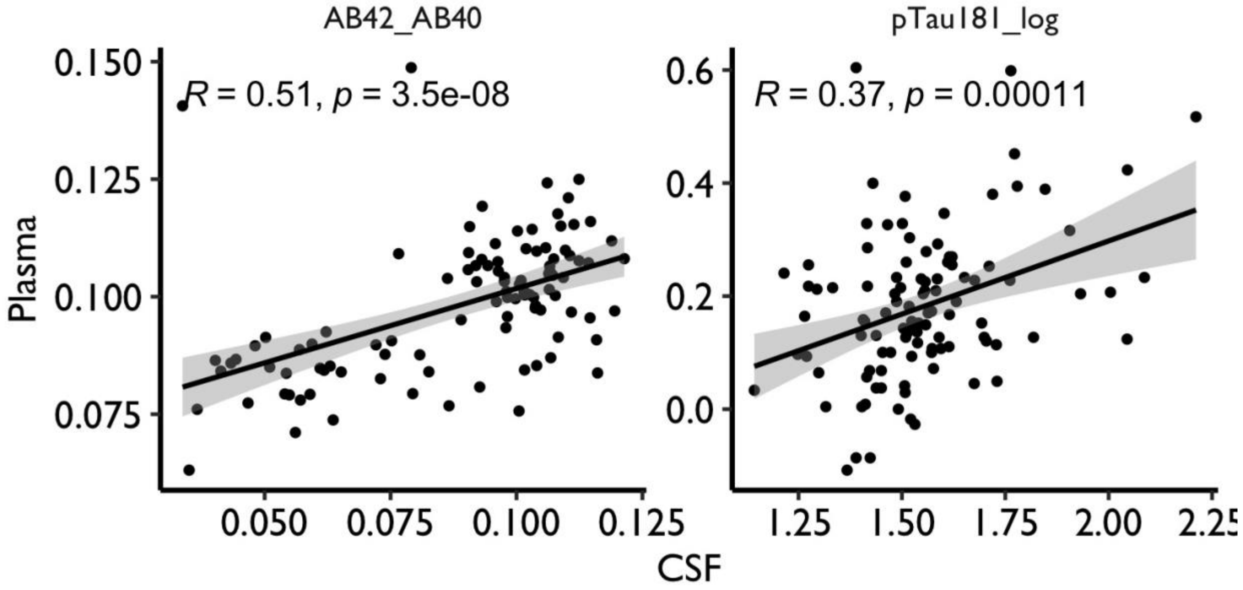


**Fig. S7. Scatterplots showing the relationship between CSF and plasma measures for (left) $A\beta_{42}/A\beta_{40}$ and (right) $pTau_{181}$.**

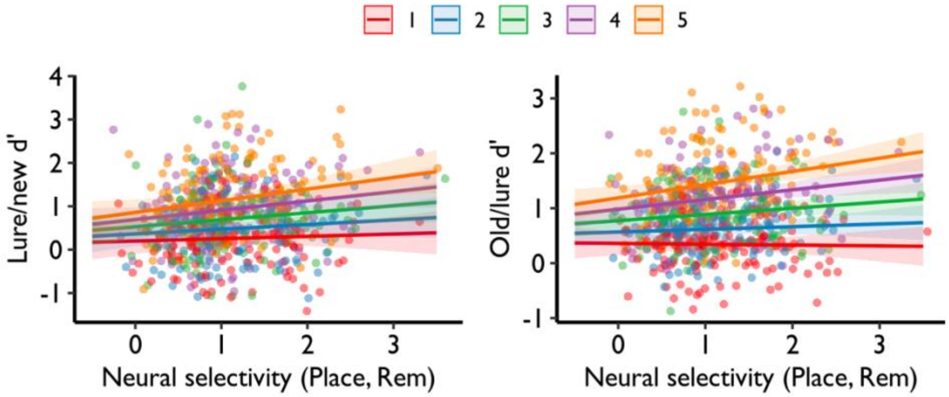


**Fig. S8. Neural selectivity interacts with target-lure similarity.** MST performance varied as a function of neural selectivity and target-lure similarity, which ranged from high (1) to low (5) similarity.

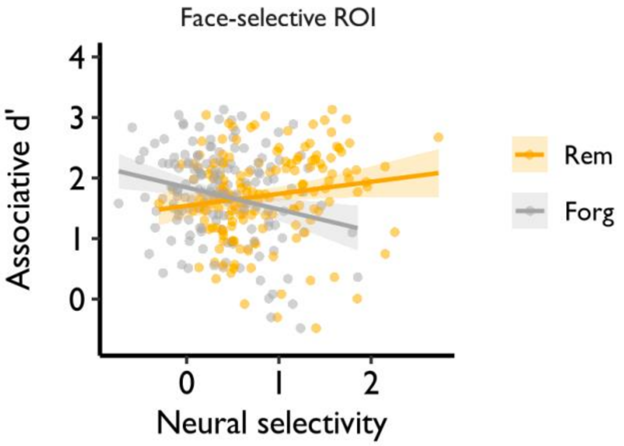


**Fig. S9. Relationship between neural selectivity in face-selective regions and overall associative *d'*.**

## REFERENCES AND NOTES


1. M. J. Kahana, A. D. Wagner, *Oxford Handbook of Human Memory* (Oxford Univ. Press, 2024); https://memory.psych.upenn.edu/Oxford_Handbook_of_Human_Memory.

2. R. Cabeza, M. Albert, S. Belleville, F. I. M. Craik, A. Duarte, C. L. Grady, U. Lindenberger, L. Nyberg, D. C. Park, P. A. Reuter-Lorenz, M. D. Rugg, J. Steffener, M. N. Rajah, Maintenance, reserve and compensation: The cognitive neuroscience of healthy ageing. *Nat. Rev. Neurosci.* **19**, 701–710 (2018).

3. V. A. Carr, J. D. Bernstein, S. E. Favila, B. K. Rutt, G. A. Kerchner, A. D. Wagner, Individual differences in associative memory among older adults explained by hippocampal subfield structure and function. *Proc. Natl. Acad. Sci. U.S.A.* **114**, 12075–12080 (2017).

4. E. C. Mormino, J. T. Kluth, C. M. Madison, G. D. Rabinovici, S. L. Baker, B. L. Miller, R. A. Koeppe, C. A. Mathis, M. W. Weiner, W. J. Jagust, Alzheimer's Disease Neuroimaging Initiative, Episodic memory loss is related to hippocampal-mediated β-amyloid deposition in elderly subjects. *Brain* **132**, 1310–1323 (2009).

5. M. Gallagher, M. T. Koh, Episodic memory on the path to Alzheimer's disease. *Curr. Opin. Neurobiol.* **21**, 929–934 (2011).

6. A. N. Trelle, V. A. Carr, E. N. Wilson, M. S. Swarovski, M. P. Hunt, T. N. Toueg, T. T. Tran, D. Channappa, N. K. Corso, M. K. Thieu, M. Jayakumar, A. Nadiadwala, W. Guo, N. J. Tanner, J. D. Bernstein, C. P. Litovsky, S. A. Guerin, A. M. Khazenzon, M. B. Harrison, B. K. Rutt, G. K. Deutsch, F. T. Chin, G. A. Davidzon, J. N. Hall, S. J. Sha, C. A. Fredericks, K. I. Andreasson, G. A. Kerchner, A. D. Wagner, E. C. Mormino, Association of CSF biomarkers with hippocampal-dependent memory in preclinical Alzheimer disease. *Neurology* **96**, e1470–e1481 (2021).

7. R. Chowdhury, M. Guitart-Masip, N. Bunzeck, R. J. Dolan, E. Düzel, Dopamine Modulates episodic memory persistence in old age. *J. Neurosci.* **32**, 14193–14204 (2012).

8. D. Tromp, A. Dufour, S. Lithfous, T. Pebayle, O. Després, Episodic memory in normal aging and Alzheimer disease: Insights from imaging and behavioral studies. *Ageing Res. Rev.* **24**, 232–262 (2015).

9. N. Kanwisher, Functional specificity in the human brain: A window into the functional architecture of the mind. *Proc. Natl. Acad. Sci. U.S.A.* **107**, 11163–11170 (2010).

10. J. D. Koen, M. D. Rugg, Neural dedifferentiation in the aging brain. *Trends Cogn. Sci.* **23**, 547–559 (2019).

11. J. D. Koen, S. Srokova, M. D. Rugg, Age-related neural dedifferentiation and cognition. *Curr. Opin. Behav. Sci.* **32**, 7–14 (2020).

12. V. R. Sommer, M. C. Sander, Contributions of representational distinctiveness and stability to memory performance and age differences. *Aging Neuropsychol. Cogn.* **29**, 443–462 (2022).

13. J. O. Goh, Functional dedifferentiation and altered connectivity in older adults: Neural accounts of cognitive aging. *Aging Dis.* **2**, 30–48 (2011).

14. D. Rakesh, K. B. Fernando, S. Mansour L, Functional dedifferentiation of the brain during healthy aging. *J. Neurophysiol.* **123**, 1279–1282 (2020).

15. S. Srokova, A. N. Z. Aktas, J. D. Koen, M. D. Rugg, Dissociative effects of age on neural differentiation at the category and item levels. *J. Neurosci.* **44**, e0959232023 (2024).

16. J. Carp, J. Park, T. A. Polk, D. C. Park, Age differences in neural distinctiveness revealed by multi-voxel pattern analysis. *Neuroimage* **56**, 736–743 (2011).

17. J. Carp, J. Park, A. Hebrank, D. C. Park, T. A. Polk, Age-related neural dedifferentiation in the motor system. *PLOS ONE* **6**, e29411 (2011).

18. J. D. Koen, N. Hauck, M. D. Rugg, The relationship between age, neural differentiation, and memory performance. *J. Neurosci.* **39**, 149–162 (2019).

19. C. Saverino, Z. Fatima, S. Sarraf, A. Oder, S. C. Strother, C. L. Grady, The associative memory deficit in aging is related to reduced selectivity of brain activity during encoding. *J. Cogn. Neurosci.* **28**, 1331–1344 (2016).

20. S. O. Murray, E. Wojciulik, Attention increases neural selectivity in the human lateral occipital complex. *Nat. Neurosci.* **7**, 70–74 (2004).

21. D.-J. Yi, T. A. Kelley, R. Marois, M. M. Chun, Attentional modulation of repetition attenuation is anatomically dissociable for scenes and faces. *Brain Res.* **1080**, 53–62 (2006).

22. J. F. Mitchell, K. A. Sundberg, J. H. Reynolds, Spatial attention decorrelates intrinsic activity fluctuations in macaque area V4. *Neuron* **63**, 879–888 (2009).

23. D. Baldauf, R. Desimone, Neural mechanisms of object-based attention. *Science* **344**, 424–427 (2014).

24. G. Xue, Q. Dong, C. Chen, Z.-L. Lu, J. A. Mumford, R. A. Poldrack, Complementary role of frontoparietal activity and cortical pattern similarity in successful episodic memory encoding. *Cereb. Cortex* **23**, 1562–1571 (2013).

25. Y. Lu, C. Wang, C. Chen, G. Xue, Spatiotemporal neural pattern similarity supports episodic memory. *Curr. Biol.* **25**, 780–785 (2015).

26. J. Sheng, L. Zhang, C. Liu, J. Liu, J. Feng, Y. Zhou, H. Hu, G. Xue, Higher-dimensional neural representations predict better episodic memory. *Sci. Adv.* **8**, eabm3829 (2022).

27. M. R. Uncapher, A. D. Wagner, Posterior parietal cortex and episodic encoding: Insights from fMRI subsequent memory effects and dual-attention theory. *Neurobiol. Learn. Mem.* **91**, 139–154 (2009).

28. M. Aly, N. B. Turk-Browne, Attention promotes episodic encoding by stabilizing hippocampal representations. *Proc. Natl. Acad. Sci. U.S.A.* **113**, E420–E429 (2016).

29. J. Sheng, S. Wang, L. Zhang, C. Liu, L. Shi, Y. Zhou, H. Hu, C. Chen, G. Xue, Intersubject similarity in neural representations underlies shared episodic memory content. *Proc. Natl. Acad. Sci. U.S.A.* **120**, e2308951120 (2023).

30. A. Gazzaley, A. C. Nobre, Top-down modulation: Bridging selective attention and working memory. *Trends Cogn. Sci.* **16**, 129–135 (2012).

31. M. Mather, L. L. Carstensen, Aging and motivated cognition: The positivity effect in attention and memory. *Trends Cogn. Sci.* **9**, 496–502 (2005).

32. P. S. Powell, J. Strunk, T. James, S. M. Polyn, A. Duarte, Decoding selective attention to context memory: An aging study. *Neuroimage* **181**, 95–107 (2018).

33. S. Mirjalili, P. Powell, J. Strunk, T. James, A. Duarte, Context memory encoding and retrieval temporal dynamics are modulated by attention across the adult lifespan. *ENeuro* **8**, ENEURO.0387-20.2020 (2021).

34. M. K. Robison, N. T. Diede, J. Nicosia, B. H. Ball, J. M. Bugg, A multimodal analysis of sustained attention in younger and older adults. *Psychol. Aging* **37**, 307–325 (2022).

35. L. Geerligs, E. Saliasi, N. M. Maurits, R. J. Renken, M. M. Lorist, Brain mechanisms underlying the effects of aging on different aspects of selective attention. *Neuroimage* **91**, 52–62 (2014).

36. A. N. Trelle, V. A. Carr, S. A. Guerin, M. K. Thieu, M. Jayakumar, W. Guo, A. Nadiadwala, N. K. Corso, M. P. Hunt, C. P. Litovsky, N. J. Tanner, G. K. Deutsch, J. D. Bernstein, M. B. Harrison, A. M. Khazenzon, J. Jiang, S. J. Sha, C. A. Fredericks, B. K. Rutt, E. C. Mormino, G. A. Kerchner, A. D. Wagner, Hippocampal and cortical mechanisms at retrieval explain variability in episodic remembering in older adults. *eLife* **9**, e55335 (2020).

37. J.-H. Kang, M. Korecka, M. J. Figurski, J. B. Toledo, K. Blennow, H. Zetterberg, T. Waligorska, M. Brylska, L. Fields, N. Shah, H. Soares, R. A. Dean, H. Vanderstichele, R. C. Petersen, P. S. Aisen, A. J. Saykin, M. W. Weiner, J. Q. Trojanowski, L. M. Shaw, Alzheimer's

Disease Neuroimaging Initiative, The Alzheimer's Disease Neuroimaging Initiative 2 Biomarker Core: A review of progress and plans. *Alzheimers Dement.* **11**, 772–791 (2015).

38. K. Blennow, M. J. de Leon, H. Zetterberg, Alzheimer's disease. *Lancet* **368**, 387–403 (2006).

39. W. J. Jagust, E. C. Mormino, Lifespan brain activity, β-amyloid, and Alzheimer's disease. *Trends Cogn. Sci.* **15**, 520–526 (2011).

40. H. Braak, E. Braak, Frequency of stages of Alzheimer-related lesions in different age categories. *Neurobiol. Aging* **18**, 351–357 (1997).

41. A. Maass, S. N. Lockhart, T. M. Harrison, R. K. Bell, T. Mellinger, K. Swinnerton, S. L. Baker, G. D. Rabinovici, W. J. Jagust, Entorhinal tau pathology, episodic memory decline, and neurodegeneration in aging. *J. Neurosci.* **38**, 530–543 (2018).

42. R. A. Sperling, E. C. Mormino, A. P. Schultz, R. A. Betensky, K. V. Papp, R. E. Amariglio, B. J. Hanseeuw, R. Buckley, J. Chhatwal, T. Hedden, G. A. Marshall, Y. T. Quiroz, N. J. Donovan, J. Jackson, J. R. Gatchel, J. S. Rabin, H. Jacobs, H.-S. Yang, M. Properzi, D. R. Kirn, D. M. Rentz, K. A. Johnson, The impact of amyloid-beta and tau on prospective cognitive decline in older individuals. *Ann. Neurol.* **85**, 181–193 (2019).

43. P. S. Insel, C. B. Young, P. S. Aisen, K. A. Johnson, R. A. Sperling, E. C. Mormino, M. C. Donohue, Tau positron emission tomography in preclinical Alzheimer's disease. *Brain* **146**, 700–711 (2023).

44. C. R. Jack, H. J. Wiste, C. G. Schwarz, V. J. Lowe, M. L. Senjem, P. Vemuri, S. D. Weigand, T. M. Therneau, D. S. Knopman, J. L. Gunter, D. T. Jones, J. Graff-Radford, K. Kantarci, R. O. Roberts, M. M. Mielke, M. M. Machulda, R. C. Petersen, Longitudinal tau PET in ageing and Alzheimer's disease. *Brain* **141**, 1517–1528 (2018).

45. V. J. Lowe, T. J. Bruinsma, H. J. Wiste, H.-K. Min, S. D. Weigand, P. Fang, M. L. Senjem, T. M. Therneau, B. F. Boeve, K. A. Josephs, M. K. Pandey, M. E. Murray, K. Kantarci, D. T. Jones, P. Vemuri, J. Graff-Radford, C. G. Schwarz, M. M. Machulda, M. M. Mielke, R. O.

Roberts, D. S. Knopman, R. C. Petersen, C. R. Jack Jr., Cross-sectional associations of tau-PET signal with cognition in cognitively unimpaired adults. *Neurology* **93**, e29–e39 (2019).

46. T. J. Betthauser, R. L. Koscik, E. M. Jonaitis, S. L. Allison, K. A. Cody, C. M. Erickson, H. A. Rowley, C. K. Stone, K. D. Mueller, L. R. Clark, C. M. Carlsson, N. A. Chin, S. Asthana, B. T. Christian, S. C. Johnson, Amyloid and tau imaging biomarkers explain cognitive decline from late middle-age. *Brain* **143**, 320–335 (2020).

47. A. Maass, D. Berron, T. M. Harrison, J. N. Adams, R. La Joie, S. Baker, T. Mellinger, R. K. Bell, K. Swinnerton, B. Inglis, Alzheimer's pathology targets distinct memory networks in the ageing brain. *Brain* **142**, 2492–2509 (2019).

48. H. Braak, K. Del Tredici, The preclinical phase of the pathological process underlying sporadic Alzheimer's disease. *Brain* **138**, 2814–2833 (2015).

49. D. M. A. Mann, J. Lincoln, P. O. Yates, J. E. Stamp, S. Toper, Changes in the monoamine containing neurones of the human CNS in senile dementia. *Br. J. Psychiatry* **136**, 533–541 (1980).

50. C. W. Berridge, B. D. Waterhouse, The locus coeruleus–noradrenergic system: Modulation of behavioral state and state-dependent cognitive processes. *Brain Res. Rev.* **42**, 33–84 (2003).

51. M. Corbetta, G. L. Shulman, Spatial neglect and attention networks. *Annu. Rev. Neurosci.* **34**, 569–599 (2011).

52. R. Xia, X. Chen, T. A. Engel, T. Moore, Common and distinct neural mechanisms of attention. *Trends Cogn. Sci.* **28**, 554–567 (2024).

53. S. Vossel, J. J. Geng, G. R. Fink, Dorsal and ventral attention systems: Distinct neural circuits but collaborative roles. *Neuroscientist* **20**, 150–159 (2014).

54. B. A. Gordon, J. M. Zacks, T. Blazey, T. L. S. Benzinger, J. C. Morris, A. M. Fagan, D. M. Holtzman, D. A. Balota, Task-evoked fMRI changes in attention networks are associated with preclinical Alzheimer's disease biomarkers. *Neurobiol. Aging* **36**, 1771–1779 (2015).

55. M. Corbetta, G. L. Shulman, Control of goal-directed and stimulus-driven attention in the brain. *Nat. Rev. Neurosci.* **3**, 201–215 (2002).

56. M. Mather, The affective neuroscience of aging. *Annu. Rev. Psychol.* **67**, 213–238 (2016).

57. S. J. Teipel, T. Meindl, M. Wagner, B. Stieltjes, S. Reuter, K.-H. Hauenstein, M. Filippi, U. Ernemann, M. F. Reiser, H. Hampel, Longitudinal changes in fiber tract integrity in healthy aging and mild cognitive impairment: A DTI follow-up study. *J. Alzheimers Dis.* **22**, 507–522 (2010).

58. H. Lemaitre, A. L. Goldman, F. Sambataro, B. A. Verchinski, A. Meyer-Lindenberg, D. R. Weinberger, V. S. Mattay, Normal age-related brain morphometric changes: Nonuniformity across cortical thickness, surface area and gray matter volume? *Neurobiol. Aging* **33**, 617.e1–617.e9 (2012).

59. C. E. Webb, D. A. Hoagey, K. M. Rodrigue, K. M. Kennedy, Frontostriatal white matter connectivity: Age differences and associations with cognition and BOLD modulation. *Neurobiol. Aging* **94**, 154–163 (2020).

60. M. K. Heckner, E. C. Cieslik, S. B. Eickhoff, J. A. Camilleri, F. Hoffstaedter, R. Langner, The aging brain and executive functions revisited: Implications from meta-analytic and functional-connectivity evidence. *J. Cogn. Neurosci.* **33**, 1716–1752 (2021).

61. Y. Li, C. Li, Q. Wu, Z. Xu, T. Kurata, S. Ohno, S. Kanazawa, K. Abe, J. Wu, Decreased resting-state connections within the visuospatial attention-related network in advanced aging. *Neurosci. Lett.* **597**, 13–18 (2015).

62. M. D. Fox, M. Corbetta, A. Z. Snyder, J. L. Vincent, M. E. Raichle, Spontaneous neuronal activity distinguishes human dorsal and ventral attention systems. *Proc. Natl. Acad. Sci. U.S.A.* **103**, 10046–10051 (2006).

63. K. A. Paller, A. D. Wagner, Observing the transformation of experience into memory. *Trends Cogn. Sci.* **6**, 93–102 (2002).

64. H. Kim, Neural activity that predicts subsequent memory and forgetting: A meta-analysis of 74 fMRI studies. *Neuroimage* **54**, 2446–2461 (2011).

65. J. Park, J. Carp, K. M. Kennedy, K. M. Rodrigue, G. N. Bischof, C.-M. Huang, J. R. Rieck, T. A. Polk, D. C. Park, Neural broadening or neural attenuation? Investigating age-related dedifferentiation in the face network in a large lifespan sample. *J. Neurosci.* **32**, 2154–2158 (2012).

66. B. Martins-Klein, I. Orlovsky, K. Heideman, Remembering past challenges to feel better today: Role of neural dedifferentiation and autobiographical integration in late-life reappraisal. *Aging Neuropsychol. Cogn.* **29**, 599–619 (2022).

67. M. W. Voss, K. I. Erickson, L. Chaddock, R. S. Prakash, S. J. Colcombe, K. S. Morris, S. Doerksen, L. Hu, E. McAuley, A. F. Kramer, Dedifferentiation in the visual cortex: An fMRI investigation of individual differences in older adults. *Brain Res.* **1244**, 121–131 (2008).

68. G. Xue, The neural representations underlying human episodic memory. *Trends Cogn. Sci.* **22**, 544–561 (2018).

69. G. Xue, Q. Dong, C. Chen, Z. Lu, J. A. Mumford, R. A. Poldrack, Greater neural pattern similarity across repetitions is associated with better memory. *Science* **330**, 97–101 (2010).

70. B. A. Kuhl, J. Rissman, A. D. Wagner, Multi-voxel patterns of visual category representation during episodic encoding are predictive of subsequent memory. *Neuropsychologia* **50**, 458–469 (2012).

71. L. Zheng, Z. Gao, X. Xiao, Z. Ye, C. Chen, G. Xue, Reduced fidelity of neural representation underlies episodic memory decline in normal aging. *Cereb. Cortex* **28**, 2283–2296 (2018).

72. S. M. Korkki, F. R. Richter, H. M. Gellersen, J. S. Simons, Reduced memory precision in older age is associated with functional and structural differences in the angular gyrus. *Neurobiol. Aging* **129**, 109–120 (2023).

73. S. Rhodes, E. E. Abbene, A. M. Meierhofer, M. Naveh-Benjamin, Age differences in the precision of memory at short and long delays. *Psychol. Aging* **35**, 1073–1089 (2020).

74. S. Srokova, P. F. Hill, J. D. Koen, D. R. King, M. D. Rugg, Neural differentiation is moderated by age in scene-selective, but not face-selective, cortical regions. *ENeuro* **7**, ENEURO.0142-20.2020 (2020).

75. D. C. Park, T. A. Polk, R. Park, M. Minear, A. Savage, M. R. Smith, Aging reduces neural specialization in ventral visual cortex. *Proc. Natl. Acad. Sci. U.S.A.* **101**, 13091–13095 (2004).

76. J. D. Koen, Age-related neural dedifferentiation for individual stimuli: An across-participant pattern similarity analysis. *Aging Neuropsychol. Cogn.* **29**, 552–576 (2022).

77. E. M. Aminoff, K. Kveraga, M. Bar, The role of the parahippocampal cortex in cognition. *Trends Cogn. Sci.* **17**, 379–390 (2013).

78. A. M. Fjell, L. McEvoy, D. Holland, A. M. Dale, K. B. Walhovd, Alzheimer's Disease Neuroimaging Initiative, What is normal in normal aging? Effects of aging, amyloid and Alzheimer’s disease on the cerebral cortex and the hippocampus. *Prog. Neurobiol.* **117**, 20–40 (2014).

79. G. Douaud, A. R. Groves, C. K. Tamnes, L. T. Westlye, E. P. Duff, A. Engvig, K. B. Walhovd, A. James, A. Gass, A. U. Monsch, P. M. Matthews, A. M. Fjell, S. M. Smith, H. Johansen-Berg, A common brain network links development, aging, and vulnerability to disease. *Proc. Natl. Acad. Sci. U.S.A.* **111**, 17648–17653 (2014).

80. O. Pascalis, D. J. Kelly, the origins of face processing in humans: Phylogeny and ontogeny. *Perspect. Psychol. Sci.* **4**, 200–209 (2009).

81. X. Yan, S. Tung, B. Fascendini, Y. D. Chen, A. M. Norcia, K. Grill-Spector, When do visual category representations emerge in infants’ brains? bioRxiv 2023.05.11.539934 [Preprint] (2024). https://doi.org/10.1101/2023.05.11.539934.

82. D. D. Dilks, F. S. Kamps, A. S. Persichetti, Three cortical scene systems and their development. *Trends Cogn. Sci.* **26**, 117–127 (2022).

83. X. J. Chai, L. Tang, J. D. Gabrieli, N. Ofen, From vision to memory: How scene-sensitive regions support episodic memory formation during child development. *Dev. Cogn. Neurosci.* **65**, 101340 (2024).

84. D. D. Dilks, Y. Jung, F. S. Kamps, The development of human cortical scene processing. *Curr. Dir. Psychol. Sci.* **32**, 479–486 (2023).

85. J. Gomez, M. A. Barnett, V. Natu, A. Mezer, N. Palomero-Gallagher, K. S. Weiner, K. Amunts, K. Zilles, K. Grill-Spector, Microstructural proliferation in human cortex is coupled with the development of face processing. *Science* **355**, 68–71 (2017).

86. M. R. Uncapher, J. B. Hutchinson, A. D. Wagner, Dissociable effects of top-down and bottom-up attention during episodic encoding. *J. Neurosci.* **31**, 12613–12628 (2011).

87. E. K. Miller, J. D. Cohen, An integrative theory of prefrontal cortex function. *Annu. Rev. Neurosci.* **24**, 167–202 (2001).

88. B. Noudoost, M. H. Chang, N. A. Steinmetz, T. Moore, Top-down control of visual attention. *Curr. Opin. Neurobiol.* **20**, 183–190 (2010).

89. B. Noudoost, T. Moore, Control of visual cortical signals by prefrontal dopamine. *Nature* **474**, 372–375 (2011).

90. B. Noudoost, T. Moore, The role of neuromodulators in selective attention. *Trends Cogn. Sci.* **15**, 585–591 (2011).

91. K. K. Schneider, A. B. Schote, J. Meyer, C. Frings, Genes of the dopaminergic system selectively modulate top-down but not bottom-up attention. *Cogn. Affect. Behav. Neurosci.* **15**, 104–116 (2015).

92. S.-C. Li, A. Rieckmann, Neuromodulation and aging: Implications of aging neuronal gain control on cognition. *Curr. Opin. Neurobiol.* **29**, 148–158 (2014).

93. S. L. Sheremata, D. C. Somers, S. Shomstein, Visual short-term memory activity in parietal lobe reflects cognitive processes beyond attentional selection. *J. Neurosci.* **38**, 1511–1519 (2018).

94. N. U. F. Dosenbach, D. A. Fair, A. L. Cohen, B. L. Schlaggar, S. E. Petersen, A dual-networks architecture of top-down control. *Trends Cogn. Sci.* **12**, 99–105 (2008).

95. P. Kok, J. F. M. Jehee, F. P. de Lange, Less is more: Expectation sharpens representations in the primary visual cortex. *Neuron* **75**, 265–270 (2012).

96. R. A. Poldrack, Is “efficiency” a useful concept in cognitive neuroscience? *Dev. Cogn. Neurosci.* **11**, 12–17 (2015).

97. B. T. Yeo, F. M. Krienen, J. Sepulcre, M. R. Sabuncu, D. Lashkari, M. Hollinshead, J. L. Roffman, J. W. Smoller, L. Zollei, J. R. Polimeni, B. Fischl, H. Liu, R. L. Buckner, The organization of the human cerebral cortex estimated by intrinsic functional connectivity. *J. Neurophysiol.* **106**, 1125–65 (2011).

98. J. C. Morris, The Clinical Dementia Rating (CDR): Current version and scoring rules. *Neurology* **43**, 2412–2414 (1993).

99. E. N. Wilson, C. B. Young, J. R. Benitez, M. S. Swarovski, I. Feinstein, M. Vandijck, Y. L. Guen, N. M. Kasireddy, M. Shahid, N. K. Corso, Q. Wang, G. Kennedy, A. N. Trelle, B. Lind, D. Channappa, M. Belnap, V. Ramirez, I. Skylar-Scott, K. Younes, M. V. Yutsis, N. L. Bastard, J. F. Quinn, C. H. van Dyck, A. Nairn, C. A. Fredericks, L. Tian, G. A. Kerchner, T. J. Montine, S. J. Sha, G. Davidzon, V. W. Henderson, F. M. Longo, M. D. Greicius, A. D. Wagner, T. Wyss-Coray, K. L. Poston, E. C. Mormino, K. I. Andreasson, Performance of a fully-automated Lumipulse plasma phospho-tau181 assay for Alzheimer’s disease. *Alzheimers Res. Ther.* **14**, 172 (2022).

100. E. N. Wilson, M. S. Swarovski, P. Linortner, M. Shahid, A. J. Zuckerman, Q. Wang, D. Channappa, P. S. Minhas, S. D. Mhatre, E. D. Plowey, J. F. Quinn, C. P. Zabetian, L. Tian, F. M. Longo, B. Cholerton, T. J. Montine, K. L. Poston, K. I. Andreasson, Soluble TREM2 is elevated in Parkinson's disease subgroups with increased CSF tau. *Brain* **143**, 932–943 (2020).

101. O. Esteban, C. J. Markiewicz, R. W. Blair, C. A. Moodie, A. I. Isik, A. Erramuzpe, J. D. Kent, M. Goncalves, E. DuPre, M. Snyder, H. Oya, S. S. Ghosh, J. Wright, J. Durnez, R. A. Poldrack, K. J. Gorgolewski, fMRIPrep: A robust preprocessing pipeline for functional MRI. *Nat. Methods* **16**, 111–116 (2019).

102. K. Gorgolewski, C. D. Burns, C. Madison, D. Clark, Y. O. Halchenko, M. L. Waskom, S. S. Ghosh, Nipype: A flexible, lightweight and extensible neuroimaging data processing framework in python. *Front. Neuroinform.* **5**, 13 (2011).

103. K. J. Gorgolewski, O. Esteban, C. J. Markiewicz, E. Ziegler, D. G. Ellis, M. P. Notter, D. Jarecka, H. Johnson, C. Burns, A. Manhães-Savio, Nipype. *Software* (2018).

104. M. F. Glasser, S. N. Sotiropoulos, J. A. Wilson, T. S. Coalson, B. Fischl, J. L. Andersson, J. Xu, S. Jbabdi, M. Webster, J. R. Polimeni, D. van Essen, M. Jenkinson, WU-Minn HCP Consortium, The minimal preprocessing pipelines for the Human Connectome project. *Neuroimage* **80**, 105–124 (2013).

105. M. Durteste, L. R. Liebi, E. Sapoval, A. Delaux, A. Arleo, S. Ramanoël, Scene-selective regions encode the vertical position of navigationally relevant information in young and older adulthood. bioRxiv 2023.10.18.562731 [Preprint] (2023). https://doi.org/10.1101/2023.10.18.562731.

106. O. Gbadeyan, J. Teng, R. S. Prakash, Predicting response time variability from task and resting-state functional connectivity in the aging brain. *Neuroimage* **250**, 118890 (2022).

107. S. Martin, D. Saur, G. Hartwigsen, Age-dependent contribution of domain-general networks to semantic cognition. *Cereb. Cortex* **32**, 870–890 (2022).

108. A. M. Winkler, G. R. Ridgway, G. Douaud, T. E. Nichols, S. M. Smith, Faster permutation inference in brain imaging. *Neuroimage* **141**, 502–516 (2016).

109. S. M. Smith, T. E. Nichols, Threshold-free cluster enhancement: Addressing problems of smoothing, threshold dependence and localisation in cluster inference. *Neuroimage* **44**, 83–98 (2009).

110. J. A. Mumford, B. O. Turner, F. G. Ashby, R. A. Poldrack, Deconvolving BOLD activation in event-related designs for multivoxel pattern classification analyses. *Neuroimage* **59**, 2636–2643 (2012).

111. A. Walther, H. Nili, N. Ejaz, A. Alink, N. Kriegeskorte, J. Diedrichsen, Reliability of dissimilarity measures for multi-voxel pattern analysis. *Neuroimage* **137**, 188–200 (2016).

112. Z. Zhen, Z. Yang, L. Huang, X. Kong, X. Wang, X. Dang, Y. Huang, Y. Song, J. Liu, Quantifying interindividual variability and asymmetry of face-selective regions: A probabilistic functional atlas. *Neuroimage* **113**, 13–25 (2015).

113. A. Schaefer, R. Kong, E. M. Gordon, T. O. Laumann, X.-N. Zuo, A. J. Holmes, S. B. Eickhoff, B. T. T. Yeo, Local-global parcellation of the human cerebral cortex from intrinsic functional connectivity MRI. *Cereb. Cortex* **28**, 3095–3114 (2018).

114. K. F. LaRocque, T. H. Davis, J. A. Mumford, R. A. Poldrack, A. D. Wagner, When multi-voxel pattern similarity and global activation are intertwined: Approaches to disentangling correlation from activation. bioRxiv 2023.05.29.542175 [Preprint] (2023). https://doi.org/10.1101/2023.05.29.542175.

115. K. F. LaRocque, M. E. Smith, V. A. Carr, N. Witthoft, K. Grill-Spector, A. D. Wagner, Global similarity and pattern separation in the human medial temporal lobe predict subsequent memory. *J. Neurosci.* **33**, 5466–5474 (2013).

116. S. M. Stark, M. A. Yassa, J. W. Lacy, C. E. L. Stark, A task to assess behavioral pattern separation (BPS) in humans: Data from healthy aging and mild cognitive impairment. *Neuropsychologia* **51**, 2442–2449 (2013).

117. G. Y. Zou, Toward using confidence intervals to compare correlations. *Psychol. Methods* **12**, 399–413 (2007).

118. J. L. Andersson, S. Skare, J. Ashburner, How to correct susceptibility distortions in spin-echo echo-planar images: Application to diffusion tensor imaging. *Neuroimage* **20**, 870–888 (2003).

119. N. J. Tustison, B. B. Avants, P. A. Cook, Y. Zheng, A. Egan, P. A. Yushkevich, J. C. Gee, N4ITK: Improved N3 bias correction. *IEEE Trans. Med. Imaging* **29**, 1310–1320 (2010).

120. B. B. Avants, C. L. Epstein, M. Grossman, J. C. Gee, Symmetric diffeomorphic image registration with cross-correlation: Evaluating automated labeling of elderly and neurodegenerative brain. *Med. Image Anal.* **12**, 26–41 (2008).

121. Y. Zhang, M. Brady, S. Smith, Segmentation of brain MR images through a hidden Markov random field model and the expectation-maximization algorithm. *IEEE Trans. Med. Imaging* **20**, 45–57 (2001).

122. A. M. Dale, B. Fischl, M. I. Sereno, Cortical surface-based analysis: I. Segmentation and surface reconstruction. *Neuroimage* **9**, 179–194 (1999).

123. G. Klein, Y. Kim, Y. Deng, J. Senellart, A. M. Rush, OpenNMT: Open-source toolkit for neural machine translation. arXiv:1701.02810 [cs.CL] (2017).

124. V. S. Fonov, A. C. Evans, R. C. McKinstry, C. R. Almli, D. L. Collins, Unbiased nonlinear average age-appropriate brain templates from birth to adulthood. *Neuroimage* **47**, S102 (2009).

125. A. C. Evans, A. L. Janke, D. L. Collins, S. Baillet, Brain templates and atlases. *Neuroimage* **62**, 911–922 (2012).

126. M. Jenkinson, P. Bannister, M. Brady, S. Smith, Improved optimization for the robust and accurate linear registration and motion correction of brain images. *Neuroimage* **17**, 825–841 (2002).

127. R. W. Cox, J. S. Hyde, Software tools for analysis and visualization of fMRI data. *NMR Biomed.* **10**, 171–178 (1997).

128. D. N. Greve, B. Fischl, Accurate and robust brain image alignment using boundary-based registration. *Neuroimage* **48**, 63–72 (2009).

129. J. D. Power, A. Mitra, T. O. Laumann, A. Z. Snyder, B. L. Schlaggar, S. E. Petersen, Methods to detect, characterize, and remove motion artifact in resting state fMRI. *Neuroimage* **84**, 320–341 (2014).

130. Y. Behzadi, K. Restom, J. Liau, T. T. Liu, A component based noise correction method (CompCor) for BOLD and perfusion based fMRI. *Neuroimage* **37**, 90–101 (2007).

131. T. D. Satterthwaite, M. A. Elliott, R. T. Gerraty, K. Ruparel, J. Loughead, M. E. Calkins, S. B. Eickhoff, H. Hakonarson, R. C. Gur, R. E. Gur, D. H. Wolf, An improved framework for confound regression and filtering for control of motion artifact in the preprocessing of resting-state functional connectivity data. *Neuroimage* **64**, 240–256 (2013).

132. R. Patriat, R. C. Reynolds, R. M. Birn, An improved model of motion-related signal changes in fMRI. *Neuroimage* **144**, 74–82 (2017).

133. C. Lanczos, Evaluation of noisy data. *SIAM J. Numer. Anal.* **1**, 76–85 (1964).

134. A. Abraham, F. Pedregosa, M. Eickenberg, P. Gervais, A. Mueller, J. Kossaifi, A. Gramfort, B. Thirion, G. Varoquaux, Machine learning for neuroimaging with scikit-learn. *Front. Neuroinform.* **8**, 14 (2014).
